# Natural chlocarbazomycins as potential adenosine A1 receptor antagonists: ligand-based and structure-based virtual screening, quantum chemical analysis and CNS MPO study

**DOI:** 10.1007/s11030-025-11446-6

**Published:** 2026-01-14

**Authors:** Emanuelle Machado Marinho, Francisco Nithael Melo Lúcio, Matheus Nunes da Rocha, Victor Moreira de Oliveira, Francisco Wagner Queiroz de Almeida-Neto, Márcia Machado Marinho, Emmanuel Silva Marinho, Pedro de Lima-Neto

**Affiliations:** 1 Department of Analytical Chemistry and Physical Chemistry, Federal University of Ceará, Fortaleza, CE Brazil; 2 Doctoral Program in Biotechnology, Northeast Biotechnology Network, State University of Ceará, Fortaleza, CE Brazil; 3 Postgraduate in Natural Sciences, Sciences and Technology Center, State University of Ceará, Fortaleza, CE Brazil; 4 Inhamuns Center for Education, Sciences and Letters, State University of Ceará, Fortaleza, CE Brazil; 5 Postgraduate Program in Veterinary Sciences, Faculty of Veterinary Medicine, State University of Ceara, Fortaleza, CE Brazil; 6 State University of Ceara, Fortaleza, CE Brazil

**Keywords:** Carbazomycins, Adenosine A_1_ receptor, Parkinson's disease, Molecular docking, Quantum chemistry.

## Abstract

Parkinson’s disease (PD) is a neurodegenerative disorder that causes irreversible damage to brain structures through neurotransmitter oxidation, leading to motor symptoms like tremors and muscle rigidity. Although existing therapies target monoamine oxidase B, recent research has highlighted​​ a correlation between adenosine A_1_ and A_2A_R receptors in inhibiting dopamine reuptake, as observed in rats. Chlocarbazomycins (CCB), carbazole derivatives with neuroprotective properties, show potential for central nervous system (CNS) therapies. This study examines the structural and bioactivity properties of four carbazomicin derivatives (CCB1-4) using quantum-level Density Functional Theory (DFT) calculations, virtual screening, and a predictive pharmacokinetics study. The results showed that different environments (water, DMSO, and chloroform) had minimal impact on the reactivity of CCB1-4 derivatives. Structure-based virtual screening revealed that the heteroaromatic nature of CCB1-4 closely resembles that of adenosine (ADN), the endogenous ligand for A_1_R receptors. Molecular docking showed that CCB3 had the highest affinity for the receptor, with a binding energy of − 8.6 kcal/mol at the ADN agonist site. Molecular dynamics simulations confirmed the stable binding of CCB3, with a free energy of − 25.9 kcal/mol, suggesting that CCB3 may act as an antagonist to ADN in A1R modulation. The results of predictive pharmacokinetic studies indicate that the compound exhibits high passive cell permeability (*P*_app, A→B_ > 10 × 10^− 6^ cm/s) and low hepatic clearance, which collectively ensure the safe activity of the compound in the CNS. These findings suggest that CCB3 has potential in PD treatment.

**Supplementary Information:**

The online version contains supplementary material available at 10.1007/s11030-025-11446-6.

## Introduction

Parkinson’s Disease (PD) is a progressive neurological condition characterised by the degeneration of specific areas of the brain, with associated motor and non-motor symptoms. Motor symptoms include muscle rigidity, tremors, and postural instability. Non-motor symptoms involve autonomic dysfunctions, such as constipation and orthostatic hypotension, which, in addition to impacting quality of life, can lead to psychiatric disorders such as depression and anxiety [1].

PD develops gradually, and its progression accelerates with advancing age, which is considered a relevant risk factor. There is a growing body of evidence to suggest that exposure to environmental factors, such as pesticides, chemical substances, and heavy metals, is associated with an increased risk of PD, as these agents can promote the destruction of dopaminergic neurons. It is therefore recommended that exposure to these environmental agents be minimized as a preventive measure [2].

Although the inhibition of Monoamine Oxidase B (MAOB) represents the most commonly reported target for the treatment of PD, recent studies have demonstrated that the antagonistic effect of adenosine A_1_ receptors (A_1_R) and adenosine A_2A_ receptors (A_2A_R), belonging to the class of G protein-coupled receptors (GPCR), exhibits a strong therapeutic correlation with the disorder [3, 4].

The antagonism of A_1_R and A_2A_R adenosine receptors, induced by the molecule 5-[5-amino-3-(4-fluorophenyl) pyrazin-2-yl]-1-isopropylpyrazin-2(1 H)-one (ASP5854), has been demonstrated to result in an increase in intracellular Ca^2+^ levels and striatal dopamine content in rats. These effects were observed after the reduction in dopamine levels resulting from the administration of 1-methyl-4-phenyl-1,2,3,6-tetrahydropyridine (MPTP) in the test species [5].

It has been demonstrated that heteroaromatic compounds containing amines, such as hydrazines and xanthines, possess alternative mechanisms for modulating AR [6, 7], particularly due to their structural similarity to endogenous ligands of these receptors, including ADN and caffeine. It is noteworthy that carbazoles, nitrogen heterocyclic compounds conjugated to aromatic systems, have been demonstrated to possess neuroprotective properties associated with neuroinflammation [8, 9], as well as reported activity against PD [10].

**Fig. 1:**
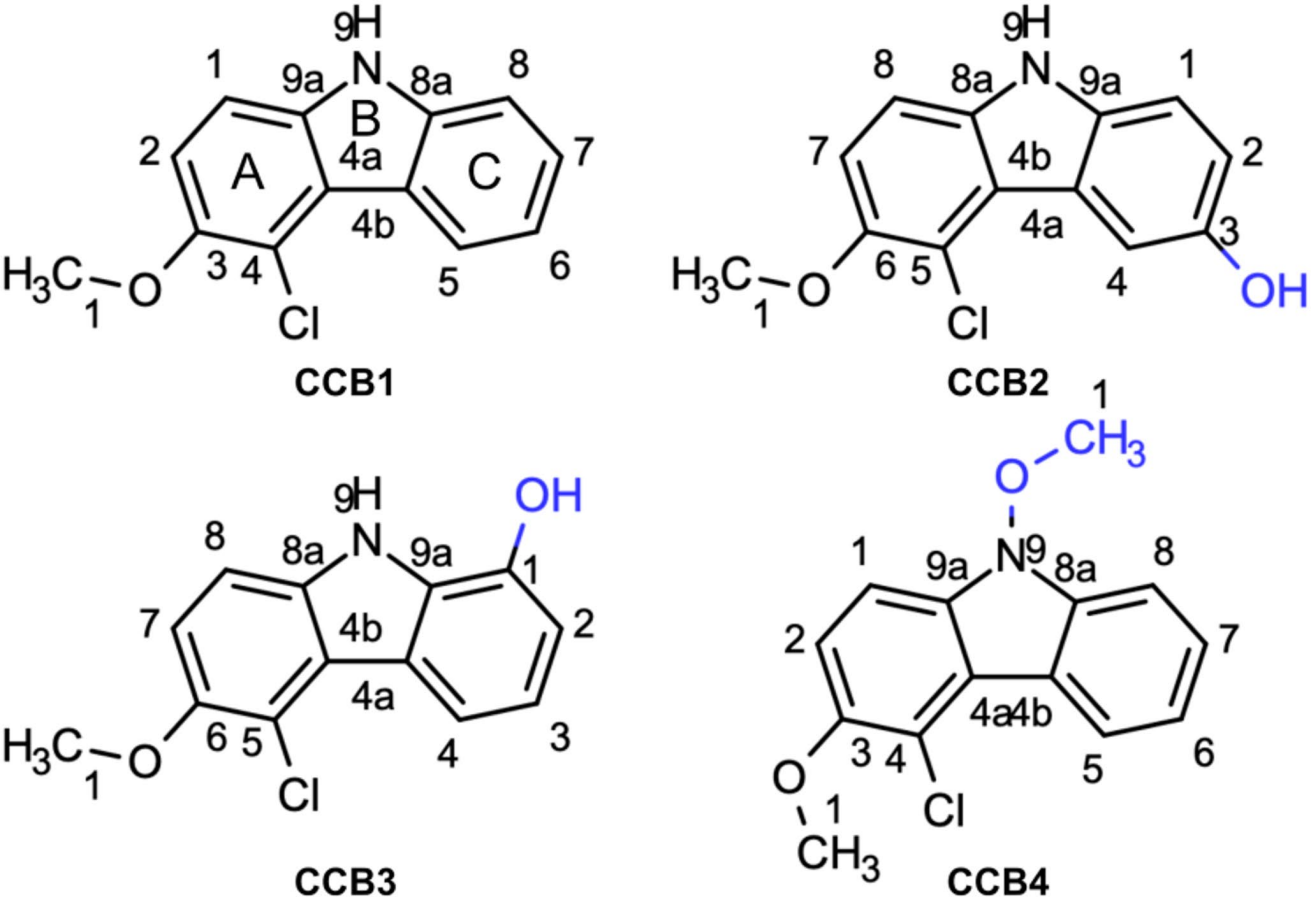
Two-dimensional representation of the chemical structure of CCB derivatives 1–4 (CCB1-4)

In a recent study, Cheng et al. (2021) [11] identified four 4-chloro- and 3-methoxy-substituted carbazole secondary metabolites, designated as chlocarbazomycins 1–4 (CCB1-4), derived from *Streptomyces diacarni* LHW51701 bacteria (Fig. 1). These metabolites demonstrated notable cytotoxic activity against human lung cancer cells, demonstrating that they are excellent cell permeants [11], an attribute that has been frequently reported in compounds with effective response to the central nervous system (CNS) [12]. These substituted fragments are directly associated with the formation of compounds that are more polar than compounds that are toxic to the CNS, thereby optimising the bioactivity of drug candidates that are modulators of GPCRs, ligands of ion channels, and enzyme inhibitors [13, 14]. These compounds are similar to nitrogenous heterocyclic compounds, but it is not clear how well this agent works compared to other A_1_R blockers, like caffeine derivatives, in treating CNS disorders.

The objective of this study is to conduct a ligand-based virtual screening (LBVS), supported by a computational approach to theoretical chemistry, of a series of CCB derivatives. This study aims to estimate the bioactivity of these compounds on A_1_R adenosine receptors for the treatment of PD.

## Methods and materials

### Quantum chemical computational calculations

For this work, all quantum chemical calculations were performed using the Density Functional Theory (DFT) method executed by the Gaussian 09 software (https://gaussian.com/glossary/g09/). To identify the three-dimensional coordinates that indicate the minimum energy of the ground state of each compound, the geometric optimization process of the structures was carried out using Becke’s three-parameter hybrid functional (B3) [15] for exchange with Lee, Yang and Parr’s gradient-corrected correlation functional (LYP) [16] and Pople’s 6–31 + G(d, p) double-ζ basis set [17]. This functional and the basis set were chosen because they correlate very well with experimental data in the study of organic molecules [18]. The optimization calculation was carried out in vacuum and with water, methanol, chloroform, and DMSO as the implicit solvent. The Integral Equation Formalism - Polarizable Continuum Model (IEF-PCM) was used in the solvation study. Vibrational frequency calculations were carried out to investigate the vibrational properties of cholocarbazomycins.

The energies of the FMO, HOMO, and LUMO were calculated to investigate global reactivity, as HOMO and LUMO energies can be used as descriptors of global reactivity [19]. All equations related to reactivity descriptors are present in the supplementary material. The energy gap (Δ*E*_gap_) is determined from the energy difference between the LUMO and HOMO orbitals (see the Supplementary material). Koopmans’ theorem enables the investigation of the ionization energy (*IE*) and the electronic affinity (*EA*) through the energies of the HOMO and LUMO orbitals, respectively. The electronegativity (χ), chemical potential (*µ*), and chemical hardness (*ŋ*) can be calculated by deriving the electronic energy of molecules (*E*) about the number of electrons (*N*) exposed to a constant external potential (*ʋ*_(r)_). Electronegativity is defined as the first-order derivative. The chemical potential is defined as the negative of electronegativity, and the chemical hardness is defined as the second-order derivative.

Using the finite difference method, the electronegativity, chemical potential, and chemical hardness can be studied in terms of the first ionization energy and the electronic affinity, which in turn can be used to determine the energies of the HOMO and LUMO orbitals. In this way, chemical softness (*S*) is expressed as the inverse of chemical hardness. Electrophilicity is associated with the electronegativity and chemical hardness of chemical compounds. The relationships between global electrophilicity index (ω) and global nucleophilicity index (ε) were also calculated, as proposed by Parr et al. [20].

All the descriptors mentioned above are related to reactivity at a global level. To study the reactivity of atomic centers, Fukui proposed a set of frontier functions that describe the electronic density of the frontier orbitals in response to changes in the total number of electrons. In this way, Fukui’s functions enable the study of the most electrophilic and nucleophilic atomic sites in molecules. Mathematically, the Fukui function is given as the first-order derivative of the electronic density (*ρ*_(r)_) about the number of electrons (*N*) in the system at a constant external potential (*v*_(r)_).

Condensed Fukui functions enable the quantitative analysis of atomic site susceptibility. To calculate these functions, the atomic population number is used to represent the amount of electron density distribution around an atom. In this way, by applying the finite distance method, we can separately study the susceptibility of compounds to nucleophilic attack (*f*_A_^+^), electrophilic attack (*f*_*A*_^*−*^) and radical attack (*f*_*A*_^*0*^). For this work, the Hirshfeld population was chosen because it performed very well in calculating the condensed Fukui functions.

The simultaneous study of electrophilic sites or nucleophilic sites can be carried out using the condensed Fukui functions and the global electrophilicity index (ω) together to calculate the double descriptor (Δ*f*) and the multiphilic descriptor (Δω).

### Target prediction screening

To predict the biological target of the compounds in question, the SwissTargetPrediction online server (http://www.swisstargetprediction.ch/) was configured to perform a similarity matrix (*S*) test between compound *i* and compound *j*. This was done by considering the input compounds CCB1-4 and more than 300,000 known bioactives that have been deposited in the ChEBML database. This was done by the correlation expressed in Eq. 1.

1 $$s=\frac{1}{{\left( {1+\frac{1}{{18}}{d_{ij}}} \right)}}$$

In this study, *d*_ij_ was calculated as the smallest Manhattan distance, derived from the 20 × 20 distances across all potential conformations of each molecule [21]. The results were expressed as a relative distribution of bioactivity across classes of therapeutic targets in the *Rattus norvegicus* organism, including GPCR receptors, enzymes, proteases, and ion channels. This was done to select the protein for molecular docking simulations, following an LBVS approach.

### Molecular Docking simulations

The three-dimensional structure of the A_1_R complexed to the endogenous agonist adenosine (ADN) and the allosteric modulator {2-amino-4-[3,5-bis(trifluoromethyl)phenyl]thiophen-3-yl}(4-chlorophenyl)methanone (MIPS521) was taken from the RCSB Protein Data Bank (PDB) repository (https://www.rcsb.org/), deposited under PDB code ID 7LD3 [22].

The protein preparation stage involves the removal of water molecules (H_2_O) and co-crystallized ligands, as well as the addition of polar hydrogens to amino acid residues and the computation of Gasteiger charges [23], which is performed using the AutoDockTools™ program (https://autodocksuite.scripps.edu/adt/). Furthermore, the grid box was defined to encompass the entire conformational space of the protein, to enable the exploration of alternative connection sites, with dimensions of x = 80 Å, y = 62 Å, and z = 54 Å, under the axes x = 95.256, y = 107.644, and z = 112.722, and a degree of exhaustiveness of 64.

The AutoDockVina™ code (https://vina.scripps.edu/) was configured to perform a cycle of 50 independent simulations, each with 20 poses, for each ligand, utilizing the Lamarckian Genetic Algorithm (LGA). The selection criterion for the optimal pose included alignment between low-affinity energy (*E*_A_ ≤ − 6.0 kcal/mol) and low root mean square deviation (RMSD ≤ 2.0 Å) [24].

### Molecular dynamics simulations

For the reproduction and MD simulations, the results of the best poses of the molecular docking simulations were used, where the complexes formed that presented the best binding free energy values (∆*G*) were selected, classified as one of the criteria for the selection and realization of the MD, among all the compounds evaluated in the molecular docking simulations, the favorable points of all the compounds against the A_1_R receptor will be considered, the parameterization and formation of the systems for the realization of the MD simulations were carried out using GROMACS 2022. 4, initially, the compounds used were properly parameterized through the application of the SwissParam online server (https://www.swissparam.ch/). This provided all the coordinates necessary for developing the molecular dynamics simulations. Next, the parameters involved in preparing the simulation systems were estimated. This process initially consisted of adding water molecules in TIP3 format [25], representing a cubic shape, followed by the addition of Na ^+^ and Cl ^−^ ions, under the direct interference of the CHARMM36 force field [26, 27]. The MD simulations were conducted using the GROMACS software.

Following the initial parameterisation stage of the proposed systems, two essential parameters were adjusted. These were the temperatures and pressures at which the system will be influenced. Regarding temperature, the V-rescale integrator was employed, calibrated to maintain a temperature of 310 K [28]. For pressure, the Parrinello-Rahman method was utilized, with the barostat calibrated to maintain a pressure of 1 bar [29] throughout the MD calculations. The simulations were conducted according to a standardised protocol, with a total simulation time of 150 ns, maintained at constant temperature and pressure. This protocol was applied to all systems evaluated, with independent and triplicate measurements taken for each system [30].

To analyze the conformational variations of each system, the variations in RMSD will be determined as shown in Eq. 2. This equation refers to the variables *x*_1_, *y*_1_, and *z*_1_, which are contained in the representation of the axes of the cartesian plane (*x*, *y*, and *z*) and are linked to a specific atom, characterized as an initial reference projection. Meanwhile, *x*_i_, *y*_i_, and *z*_i_ represent the coordinates of the same atom compared to a second moment after the molecular dynamics simulation. These calculations demonstrate how the entire system varies over time, allowing us to observe how each complex behaves and indicating whether the system exhibits indices associated with stability [31].

2 $${\mathrm{RMSD}}=\sqrt {\frac{1}{N}\mathop \sum \limits_{{i=1}}^{N} \left[ {{{\left( {{x_1} - {x_i}} \right)}^2}+{{\left( {{y_1} - {y_i}} \right)}^2}+{{\left( {{z_1} - {z_i}} \right)}^2}} \right]~} $$

In order to simulate the variations in flexibility or rigidity presented by the amino acid residues in the biological receptor, the variations in RMSF fluctuations (Root Mean Square Fluctuation), presented in Eq. 3, will be considered. The reference terms *x*_ref_, *y*_ref_, *z*_ref_ are defined for each atom present in the system and standardized as the starting point. On the other hand, the coordinates *x*_i_, *y*_i_, *z*_i_ denote the instantaneous position based on the moment in time t, as determined by molecular dynamics simulation. The results demonstrate the displacement of all the receptor atoms at two distinct moments during the simulation, designated as the initial and final states [32]. Regarding the RMSF, the behavior of the systems was evaluated over 150 ns, during which the flexibility or rigidity of the systems was considered to assess the potential interference caused by the presence of the ligands introduced into the simulated systems.

3 $$RMS{F_{atom}}=\sqrt {\frac{1}{T}\mathop \sum \limits_{{i=1}}^{T} \left[ {{{\left( {{x_{ref}} - {x_i}} \right)}^2}+{{\left( {{y_{ref}} - {y_i}} \right)}^2}+{{\left( {{z_{ref}} - {z_i}} \right)}^2}} \right]} $$

The MM/GBSA values were calculated and inserted in Eq. 4, where the possible contributions of the terms ΔG_bind_ (binding free energy), E_vdW_ (van der Waals interaction energy), E_ele_ (electrostatic interactions), G_GB_ (polar solvation energy), G_SA_ (non-polar solvation), and -TΔS (entropic contribution due to temperature application) are observed. For each complex formed between the target receptor A_1_R and the possible compounds inserted into the specific cavities of the receptor. The results consist of MD simulations, through the respective trajectory files for each complex, and MM/GBSA calculations were implemented to estimate the value of the binding free energy existing between the receptor and the ligand [33].

4 $$\Delta {G_{bind}}={E_{vdW}}+{E_{ele}}+{G_{GB}}+{G_{SA}} - T\Delta S$$

The objective of MD prediction simulations is to demonstrate how the receptor will behave when introduced into a medium comprising water molecules and ions, with potential variables such as temperature and pressure maintained at their specified values. Consequently, the simulations can identify specific variations for each system, thereby highlighting potential indicators of stability or instability. This directly corroborates the inhibition potential of the biological receptor.

Studies that focus on the application of Principal Component Analysis (PCA) aim to highlight the potentially most relevant collective movements present in the individually evaluated systems. In order to calculate the respective structures, it was necessary to make alignment adjustments with the premise of eliminating global translation and rotation, ensuring that only internal fluctuations were analysed. The eigenvalues obtained by the calculations indicated the respective contributions of each total movement, while the eigenvectors were found to be linked to the main direction related to molecular dynamics [34].

### MPO-based PAMPA prediction

In the context of the predictive pharmacokinetics study, a Central Nervous System Multiparameter Optimization (CNS MPO) approach was employed, as proposed by da Rocha et al. (2023) [35]. The optimised chemical structures of the CCB1-4 derivatives were subjected to Molecular Lipophilicity Potential (MLP) surface analysis utilizing the open-source version of the Python Molecular Graphics (PyMOL^®^) program, as illustrated in Eq. 5:

5 $$MLP=\mathop \sum \limits_{{i=1}}^{N} {F_i} \cdot f\left( {{d_{ik}}} \right)$$

where *N* represents the number of molecular fragments present in the compounds, *F* denotes the lipophilicity index assigned to each fragment *i*, and *f*(*d*) signifies the spatial distance function (*k*) between fragments *i* [36]. The surface generated is associated with the MEP, as expressed by Eq. 6:

6 $$\Pi =\frac{1}{m}\mathop \sum \limits_{{i=1}}^{m} \left| {{V_{\left( {{r_i}} \right)}} - \tilde {V}} \right|$$

Where *m* is the number of molecular fragments, *V*(*r*_i_) is the MEP of a molecular fragment *r*_i,_ and *Ṽ* is the MEP of the total molecular surface. At the same time, the expression Π identifies the PSA in their quantitative atom neighborhoods [37]. The results were related to the lipophilicity descriptors (logP and logD at pH 7.4) and TPSA, calculated using the ADMETlab 3.0 online server (https://admetlab3.scbdd.com/).

The physicochemical properties of the CCB1-4 derivatives were calculated using the ADMETlab 3.0 predictor [38] and applied to the CNS MPO druglikeness scoring criteria (Pfizer Inc.) as shown in Eq. 7:

7 $$D=\mathop \sum \limits_{{i=1}}^{M} {w_k}{T_k}\left( {x_{k}^{0}} \right)$$

Where *w* is the weighting factor assigned to each physicochemical property *k* calculated within the ideality spectrum formed by the desirability functions (*D*), which consider the thresholds *T*(*x*) formed by intrinsic lipophilicity (logP) ≤ 3, buffer lipophilicity (logD at pH 7.4) ≤ 2, molecular weight (MW) ≤ 360 g/mol, Topological Polar Surface Area (TPSA) 40–90 Å^2^, H-bond donors (HBD) ≤ 1, basic pKa ≤ 8 (*M* = 6) [14], graphically inspected in the chemical intelligence software OSIRIS DataWarrior^®^ v06. 01.00, Openchemlib© [39]. The sum results in a druglikeness score ranging from 0 to 6, according to pharmacokinetic feasibility, which is convenient for the CNS.

The results were associated with the Parallel Artificial Membrane Permeability Assay (PAMPA) parameters predicted by the consensus test between the Deep-PK (https://biosig.lab.uq.edu.au/deeppk/) and ADMETlab 3.0 (https://admetlab3.scbdd.com/) platforms, which include cell effective permeability (*P*_app, A→B_) in adenocarcinoma colorectal (Caco-2) and Madin-Darby Canine Kidney (MDCK) cell lines, P-glycoprotein (P-gp) substrate, hepatic clearance (*CL*_int, u_), Plasma Protein Binding (PPB) and blood-brain barrier permeability (BBB), for estimation of intestinal absorption and activity in the CNS by cell permeability and clearance [40]. At the same time, metabolism site prediction and toxicity endpoints were predicted by the training set of the machine learning model of the ADMETlab 3.0 (https://admetlab3.scbdd.com/) and XenoSite (https://xenosite.org/) online servers, identifying major cytochrome P450 (CYP450) substrates (2C9, 2D6 and 3A4) and toxicophores causing organic toxicity [41].

## Results and discussion

### Quantum chemical calculations

#### Geometrical optimization

**Fig. 2:**
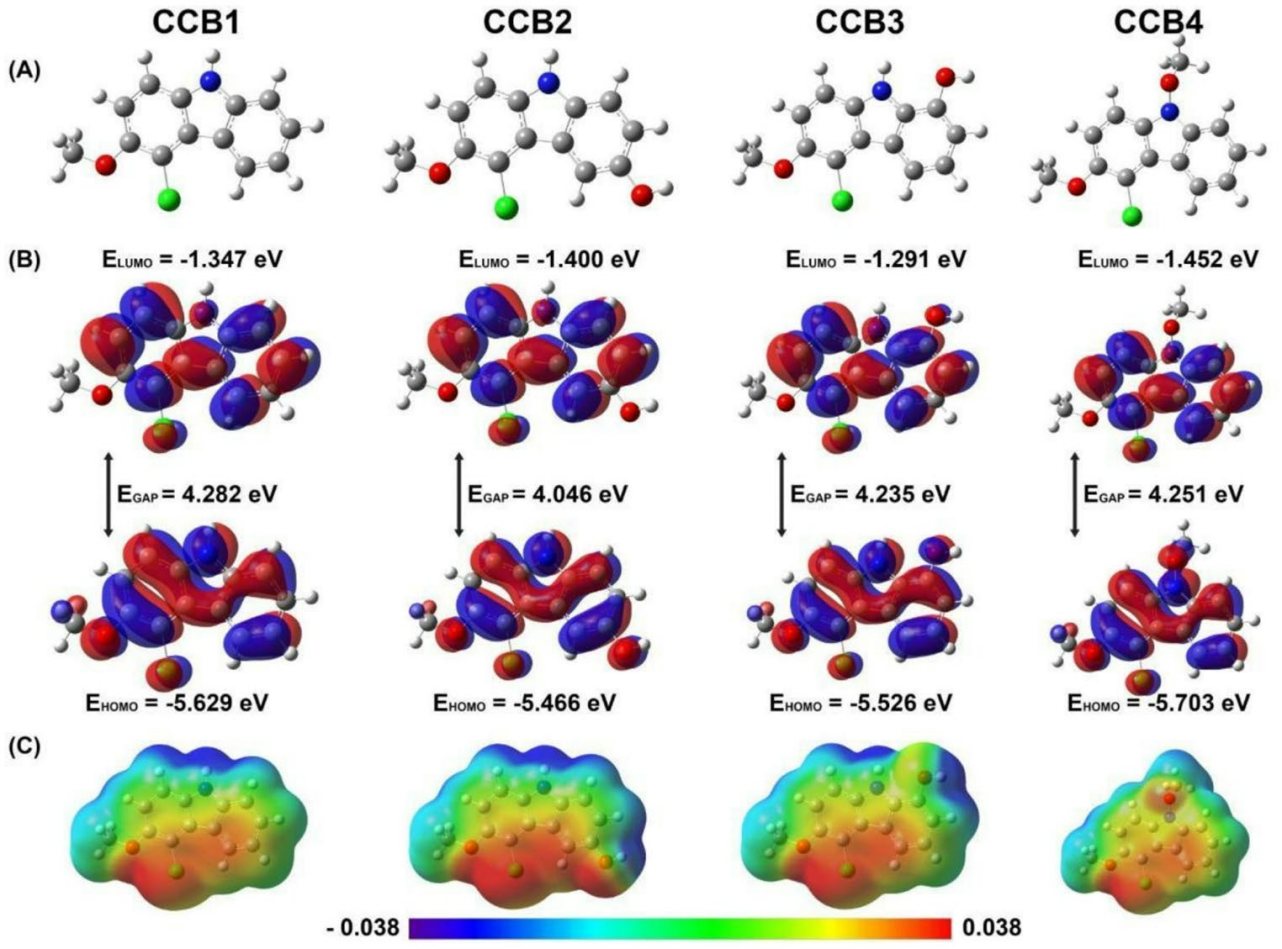
**A** Three-dimensional representation of the optimized structure of CCB1-4 derivatives in Water. **B** HOMO and LUMO frontier orbitals and gap energy (*E*_GAP_) of CCB1-4 derivatives. **C** Molecular Electrostatic Potential calculated in water for clocarbazomicins CCB1-4

Carbazoles are heterocyclic aromatic organic compounds that have a tricyclic structure, containing two benzene rings each fused to one side of a five-membered heteroatom nitrogen-containing ring. CCB1–4 have the methoxy group (OCH_3_) linked to the C5 position of the benzene ring A, and the chlorine atom is related to the C6 position, also on the benzene ring A. The structural difference between the carbazole compounds lies in the presence of the hydroxyl group (OH) linked at the C14 and C13 positions of the benzene ring A, respectively, for CCB2 and CCB3. CCB4 has the methoxy group (OCH_3_) substituted on the nitrogen of the heteroatom ring. The optimized structures of the CCB1–4 (Fig. 2A) show planar conformations, due to their rigid structures formed by three fused aromatic rings, where the planarity can be highlighted by the angles C3–C2–N11–C10 of ≈ − 179.99° and C6–C1–C9–1C12 of ≈ − 0.05° in all the compounds. The geometric data in the simulated environments showed no significant variations.

#### Electronic properties

The Highest Occupied Molecular Orbital (HOMO) is associated with the electron-donating capacity, while the Lowest Unoccupied Molecular Orbital (LUMO) is associated with the electron-accepting capacity. Moreover, these molecular orbitals represent the boundary between the ground state and the excited state, where the energy gap between these orbitals signifies the minimum excitation energy required for the electronic transition from the ground state (HOMO) to the first excited state (LUMO). Thus, investigating these properties can provide valuable insights for determining the kinetic stability and chemical reactivity of potentially bioactive compounds. The isosurfaces of the frontier molecular orbitals, HOMO and LUMO, of CCB were plotted, and the energy values of each orbital were measured. Figure 2B shows the isodensities of CCB1–4 calculated in water, where we can observe that the isodensity of the HOMO molecular orbital is delocalized across the entire molecular structure of all CCB, primarily in the π-bonds of the fused rings. The comprehension of these parameters has the potential to yield proposals for charge transfer mechanisms in protein-ligand interactions [42].

The interpretation of molecular orbital energies can be simplified significantly by using Koopmans’ Theorem. According to the theorem, the LUMO energy approximates the electron affinity. In contrast, the HOMO energy of a neutral molecule can be considered an approximation of the ionization energy of the molecule [43]. This association enables the direct relationship between the energies of the HOMO and LUMO orbits and chemical descriptors, such as a compound’s tendency to lose or gain electrons, facilitating the theoretical analysis of chemical reactivity.

Table S1 (supplementary material) shows the values of the global chemical reactivity descriptors calculated for CCB1–4 in chloroform, DMSO, methanol, and water. First, analyzing the descriptors in water, CCB2 has the valence electron density most susceptible to donation, as it presents the lowest value for ionization potential (CCB2 *IE*_water_ = 5.466 eV). CCB4, on the other hand, has the valence electron density least susceptible to donation, as it presents the highest ionization potential value (CCB4 *IE*_water_= 5.703 eV). Regarding the accommodation of an extra negative charge, CCB4 shows the highest electron affinity value (CCB4 *EA*_water_ = 1.452 eV), indicating that it can better accommodate the additional negative charge. Conversely, CCB3 tends to accommodate the extra negative charge less effectively than the other CCBs, as it presents the lowest electron affinity value (CCB3 *EA*_water_= 1.291 eV). In terms of kinetic stability, CCB1, CCB3, and CCB4 exhibited similar behavior, whereas CCB2 showed the lowest value. This suggests that CCB2 has lower kinetic stability, as it requires less energy for the electronic transition from the HOMO molecular orbital to the LUMO (gap Δ*E*_water_ = 4.066 eV).

These results suggest that CCB2 has greater electron donation potential but may be less stable. Conversely, CCB3 exhibits a reduced capacity to accommodate additional electrons received during intermolecular interactions, and may interact with biological receptors predominantly through weaker interactions, including hydrophobic interactions and conventional van der Waals interactions. A delocalization of electron density is observed in the HOMO and LUMO orbitals at the π* position of the aromatic structure of the carbazole ring, due to the mesomeric effect present in all fused rings that constitute the carbazole ring (Fig. 2B). This effect favours the formation of interactions and π-stacking in ligand-receptor interactions [44–46].

Regarding electrophilic and nucleophilic behavior, all CCB showed higher values for electrophilic potential, with CCB4 presenting the highest value, and the analysis of HOMO and LUMO suggests that this factor is independent of the solvent. This implies that, for molecular docking simulations, the compounds should retain their electronic properties. Moreover, the energy gap also exhibited little difference across solvents, indicating that the reactivity, in terms of electronic transitions, remains stable in different solvents. The electronegativity index and electron affinity also showed minimal variations between solvents, further reinforcing the idea that CCB1–4 maintain a stable electronic nature in different environments. The global chemical hardness remains practically constant, as does the global chemical softness. This indicates that the molecules exhibit a consistent response to chemical interactions, regardless of the solvent. The variation in electrophilicity and nucleophilicity indices is minimal, suggesting that different solvents do not significantly influence the electrophilic or nucleophilic character. Thus, the data show that CCB exhibits consistent electronic and energetic stability across different solvent environments, with slight variations likely related to the solvent’s polarity.

#### Fukui condensed functions

The Fukui functions (*f*_A_^+^ for nucleophilic attack and *f*_A_^−^for electrophilic attack), as well as the double descriptor (Δ*f*) and the multiphilic descriptor (Δω), were used to investigate the local reactivity of the CCB derivatives. In general, all derivatives showed similar reactivity profiles, with specific carbon and heteroatom positions being consistently highlighted as the most reactive sites (Figs. S6–S9, Supplementary Material). However, the different substituents present in the molecules modulate these profiles in a systematic manner. In CCB1, which has no additional donor or acceptor groups other than the methoxy at C1, the Fukui functions indicate a more uniform distribution of susceptibility to nucleophilic and electrophilic attacks. In the CCB2 and CCB3 derivatives, the presence of hydroxyl groups increases the local electron density through resonance effects and the possibility of hydrogen-bonding interactions. This is reflected, for example, in the increased susceptibility to electrophilic attack at C1 (CCB3) and C3 (CCB2). Finally, the methoxy substitution at N9 observed in CCB4 significantly alters the electron distribution in the heteroaromatic system, promoting greater delocalization of electron density to the adjacent π-system. Consequently, this derivative displays a more pronounced differentiation between nucleophilic and electrophilic centers, suggesting greater potential for selective interactions in the receptor active site. In this sense, the results show that, although the four derivatives share a conserved pattern of reactive sites, the presence and nature of the substituents subtly but significantly adjust the magnitude and distribution of local reactivity.

#### Molecular electrostatic potential (MEP)

The Molecular Electrostatic Potential (MEP) is a crucial tool for understanding the distribution of electronic density in molecules of interest and how these distributions can affect intermolecular interactions. The MEP surfaces for the CCB, computed at the B3LYP/6-311 + + G(d, p) level of theory, are shown in Fig. S3 for the solvents chloroform, DMSO, and methanol, and in Fig. 2C for water. The MEP surfaces display color gradients ranging from blue (low electronic density), through green (neutral zone), to yellow, orange, and red (high electronic density). Derivative CCB1 exhibits regions of intense red color over the oxygen and chlorine atoms due to the high electronegativity of these atoms, and blue color on the hydrogen atoms, with the highest intensity observed for H3. A green region represents the carbon skeleton, and the presence of electron-withdrawing groups via inductive effects in ring A reduces the electronic density in this region, displaying a yellow surface.

In contrast, ring C shows a yellowish-red surface. Isomers CCB2 and CCB3 show an electronic distribution similar to derivative CCB1, differing only in the behavior of O2. For CCB2, O2 exhibits a region of high electronic density, displaying intense red coloration, whereas in derivative CCB3, atom O2 shows lower electronic density with light yellow coloration. This discrepancy can be attributed to hydrogen bonding between O2 and H3 in CCB3. For derivative CCB4, minor differences in electronic density distribution are observed, with less intense colorations in regions of both high and low electronic density compared to the other derivatives. When analyzing different environments, the dielectric constant affects the distribution of electronic density. Thus, a higher dielectric constant leads to greater separation of partial charges and increased differences in electronic density distribution in the analyzed molecules.

### Ligand-based virtual screening (LBVS) and target prediction

**Fig. 3:**
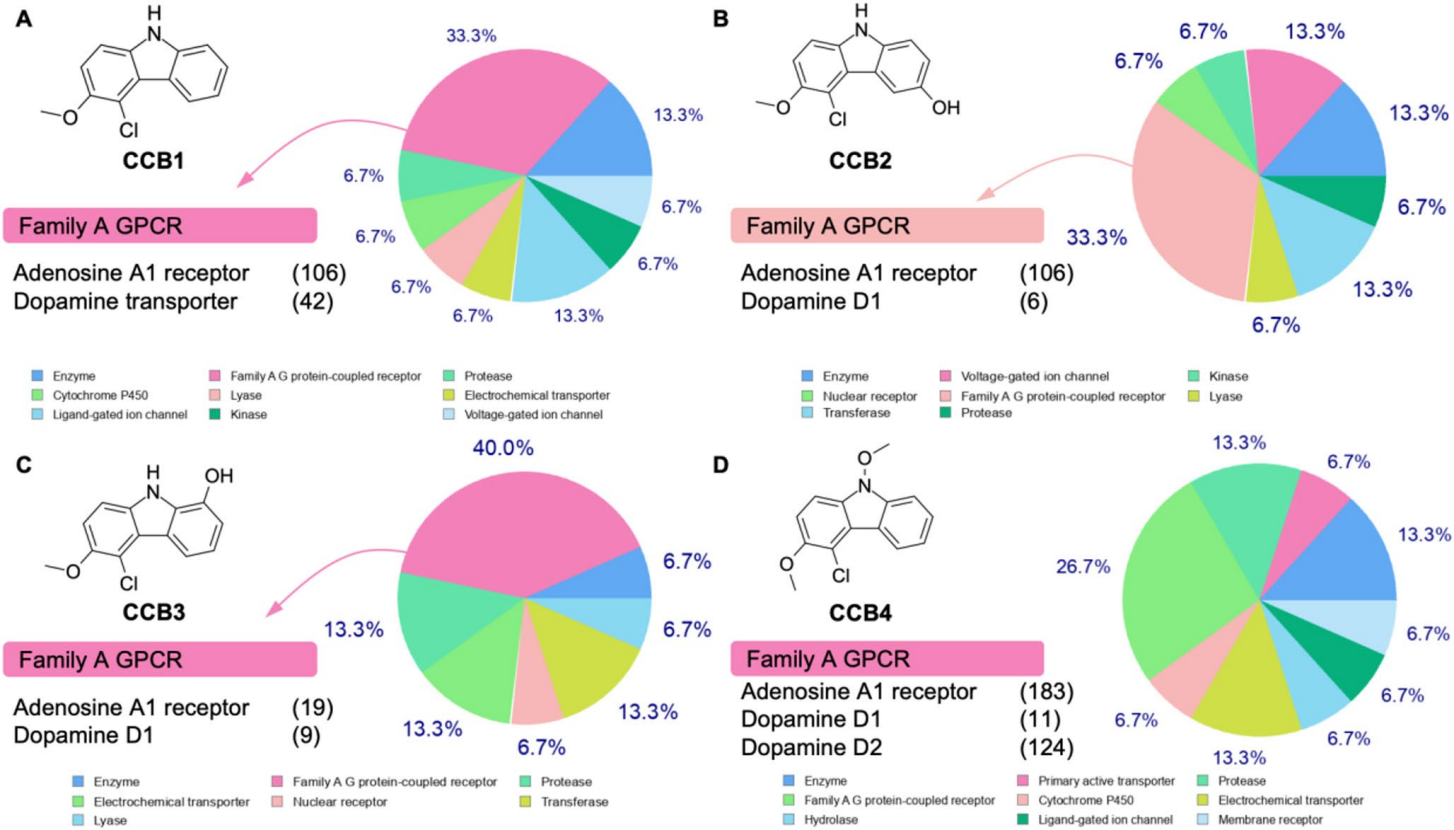
**A** Affinity energy (*E*_A_) obtained at the end of the molecular docking simulations, **B** docking of the CCB1-4 derivatives concerning the ADN in the A_1_R receptor, and details of the ligand-receptor interactions of **C** ADN and **D** CCB1-4 derivatives

An LBVS approach was employed to predict biological binding targets [47], with the results presented in Fig. 3. The target prediction test utilizes a machine learning model based on the structural similarity of the input compounds with known bioactives deposited in the ChEMBL database [48]. Overall, this scaffold exhibited a substantial degree of structural similarity with G protein-coupled receptor (GPCR) modulators of the A family, which are recognized as membrane receptors that are extensively distributed throughout the CNS (Fig. 3). In this particular case, it was possible to highlight CCB3, which demonstrated the highest spectrum of similarity among the protein classes evaluated (Fig. 3c), with 40% of its interactions involving GPCRs.

These results suggest the potential for dual modulatory activity associated with the involvement of the A_1_R and D1 dopamine receptor mechanisms, which exhibited more pronounced effects in the CCB1 (Fig. 3a) and CCB4 (Fig. 3d) derivatives. The CCB1 compound exhibited structural similarity with 42 dopamine transporter modulators (Fig. 3a), while CCB4 demonstrated structural similarity with 11 dopamine D1 receptor ligands, and 124 dopamine D2 receptor ligands, although it exhibited greater similarity with A_1_R ligands (183), indicating a higher degree of expression of dual activity in comparison to the other CCB derivatives (Fig. 3d). The hydroxylation of the CCB2 structure, in relation to the CCB1 structure, considerably reduced the susceptibility of this scaffold to interact with dopamine receptors (Fig. 3b). A recurrent outcome of this research is the observation of a propensity for the compounds to demonstrate selectivity for A_1_R receptors. This phenomenon results in the selection of the target for molecular docking simulations, which are utilized to facilitate a more in-depth investigation of their biological potential.

The results suggest that the chlocarbazomycins scaffold exhibits high structural specificity for GPCRs, which may lead to future molecular recognition research. This is because heteroaromatic compounds can intercalate with aromatic and alkyl side chain amino acid residues. These residues characterize the bases of hydrophobic cavities in membrane receptors [45, 46].

This preliminary estimate is of critical importance, as dual activity involving adenosine and dopamine receptors has been reported among compounds that have demonstrated promising activity in the reduction of the progression of Parkinson’s disease [49]. These results offer important insights regarding the potential of CCB derivatives to act through both pathways.

### Molecular Docking simulations with the A_1_R receptor

**Fig. 4:**
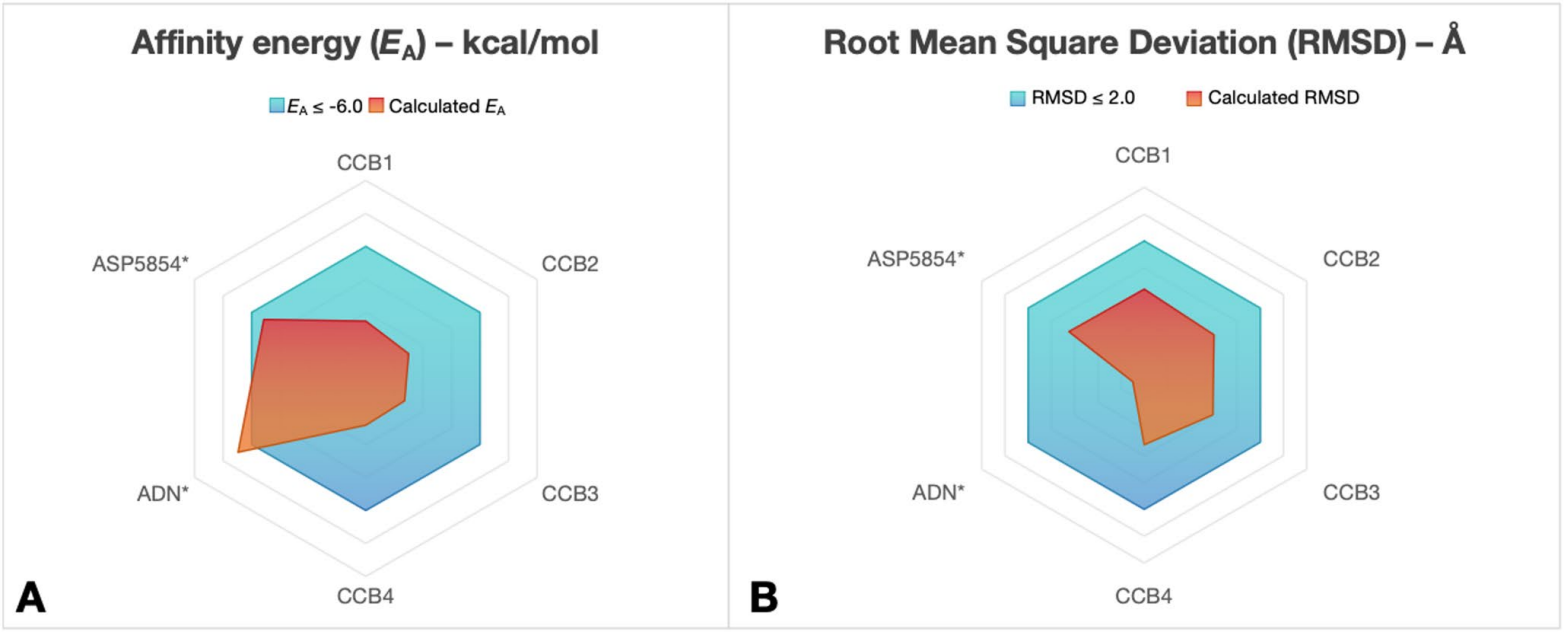
**A** Radar chart with plotted affinity energy (EA) values relative to the ideal threshold (blue area) for compounds CCB1-4, *agonist ADN, and *antagonist ASP5854 (orange area) and **B** Root Mean Square Deviation (RMSD) relative to the ideal threshold (blue area) for compounds CCB1-4, *agonist ADN, and *antagonist ASP5854 (orange area)

**Fig. 5:**
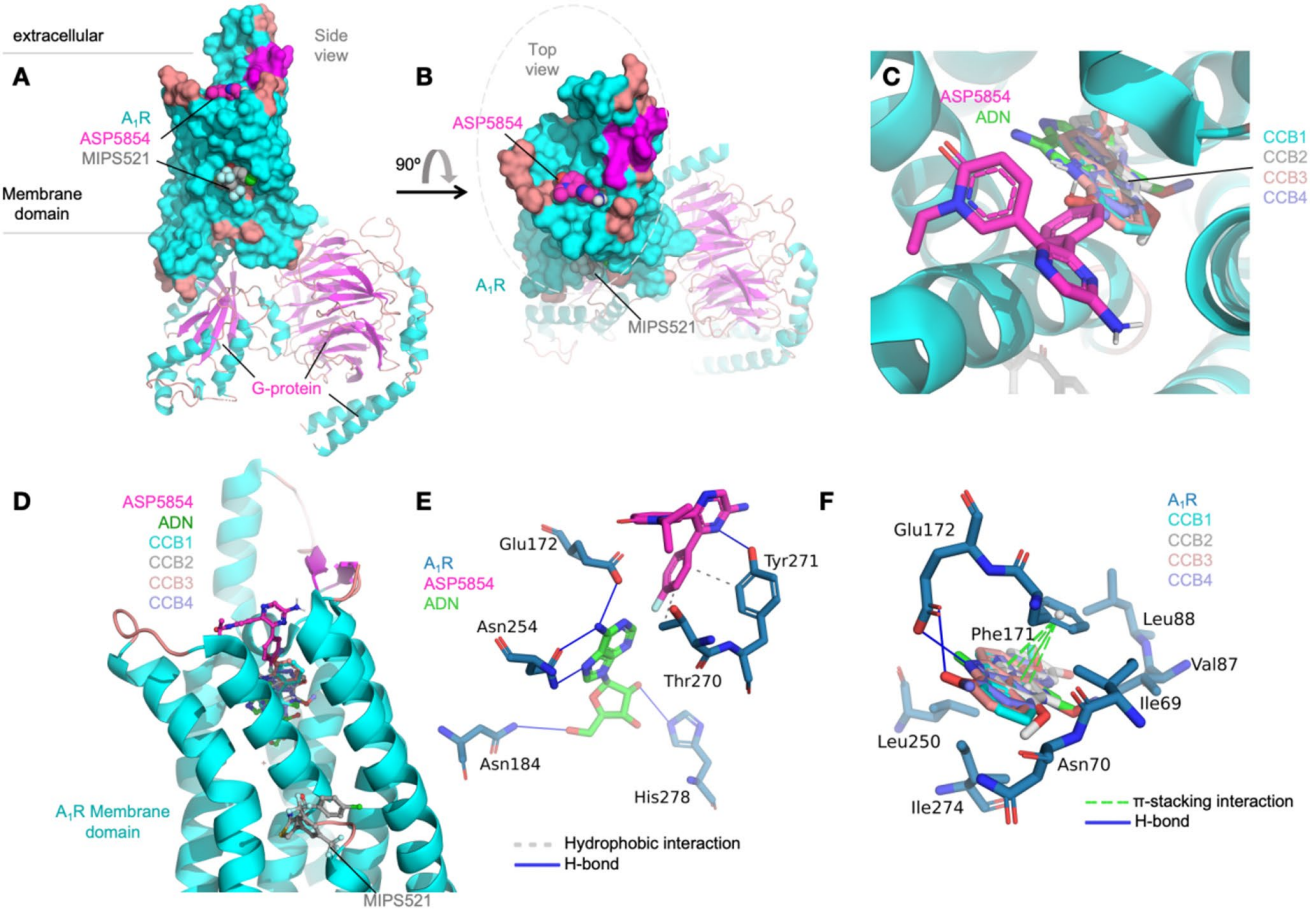
**A** Affinity energy (*E*_A_) obtained at the end of the molecular docking simulations, **B** docking of the CCB1-4 derivatives concerning the ADN in the A_1_R receptor, and details of the ligand-receptor interactions of **C** ADN and **D** CCB1-4 derivatives

This study evaluated the anti-Parkinsonian effect of CCB1-4 derivatives using molecular docking simulations, targeting the A_1_R receptor identified through ligand-based virtual screening (Table 1). Upon completion of the 50 independent simulations, each comprising 20 poses, it became evident that the CCB analogues exhibited a high degree of binding affinity with the receptor’s active site, with an order of affinity energy (*E*_A_) less than − 8.0 kcal/mol (Fig. 4), which is within the optimal threshold for CNS-active compounds that are penetrating the blood-brain barrier (*E*_A_ ≤ − 6.0 kcal/mol) [50]. This indicates that the compounds exhibit excellent specificity for the binding site of the membrane domain of the A_1_R receptor (Fig. 5A), as well as a greater affinity for the receptor compared to the endogenous ligand adenosine (ADN) [50]. The antagonist ASP5854 has also been identified within this binding domain (Fig. 5B), suggesting that the effect of the endogenous substrate ADN is counteracted by the binding of the antagonist. This observation indicates that CCB derivatives may possess A_1_R modulatory activity analogous to that of ADN [51]. This effect is attributable to the fact that CCB derivatives complex in the same binding domain as ADN (Fig. 5C).

In this instance, it is noteworthy to mention the *E*_A_ of the CCB3 compound, which was calculated to be approximately − 8.6 kcal/mol. This value is higher (more negative) than that of the antagonist ASP5854, whose calculated value is − 6.428 kcal/mol (Table 1). This indicates that CCB3 has a stronger affinity for the A_1_R receptor. The biological implications of this result suggest that the presence of ASP5854 may reverse the modulatory effect of CCB3 on the A_1_R receptor, raising the hypothesis that CCB3 may bind to the orthosteric site of the receptor’s transmembrane binding domain (Fig. 5A), similar to the endogenous substrate ADN (Fig. 5A) [52].

The simulations, with the best poses selected, were conducted within a statistical threshold formed by a RMSD of less than 2.0 Å (Fig. 4), indicating a low mean square deviation. The CCB derivatives met this threshold [53], which complexed in the same binding site as ADN, located in the transmembrane domain (Fig. 5C). Furthermore, it was observed that the compounds can perform non-competitive modulation in relation to MIPS521, a positive allosteric modulator of the A_1_R receptor (Fig. 5D). In experimental tests, this modulator reversed the binding of compounds that showed a positive response against pain by inhibiting A_1_R receptors [22], indicating a possible applicability when experimentally testing CCB derivatives as A_1_R modulators.

The molecular recognition analyses revealed that the interactions between the endogenous ADN ligand and the A_1_R receptor are predominantly hydrogen-based, with the nitrogenous base and the primary amine group (-NH_2_) engaging with the polar portion of the The side chains of the Glu172 and Asn254 residues were also observed to interact with the polar portion of the Asn184 and His278 residues (Fig. 5E) [22]. It is noteworthy that the CCB1-4 derivatives exhibited specific interactions with ADN at the amino acid residues of the A_1_R active site, including hydrogen interactions with the Glu172 residue (Fig. 5F). This highlights that the aromatic nature of the compounds favoured the formation of π-stacking interactions with the aromatic portion of the Phe171 residue (Fig. 5F).

**Fig. 6:**
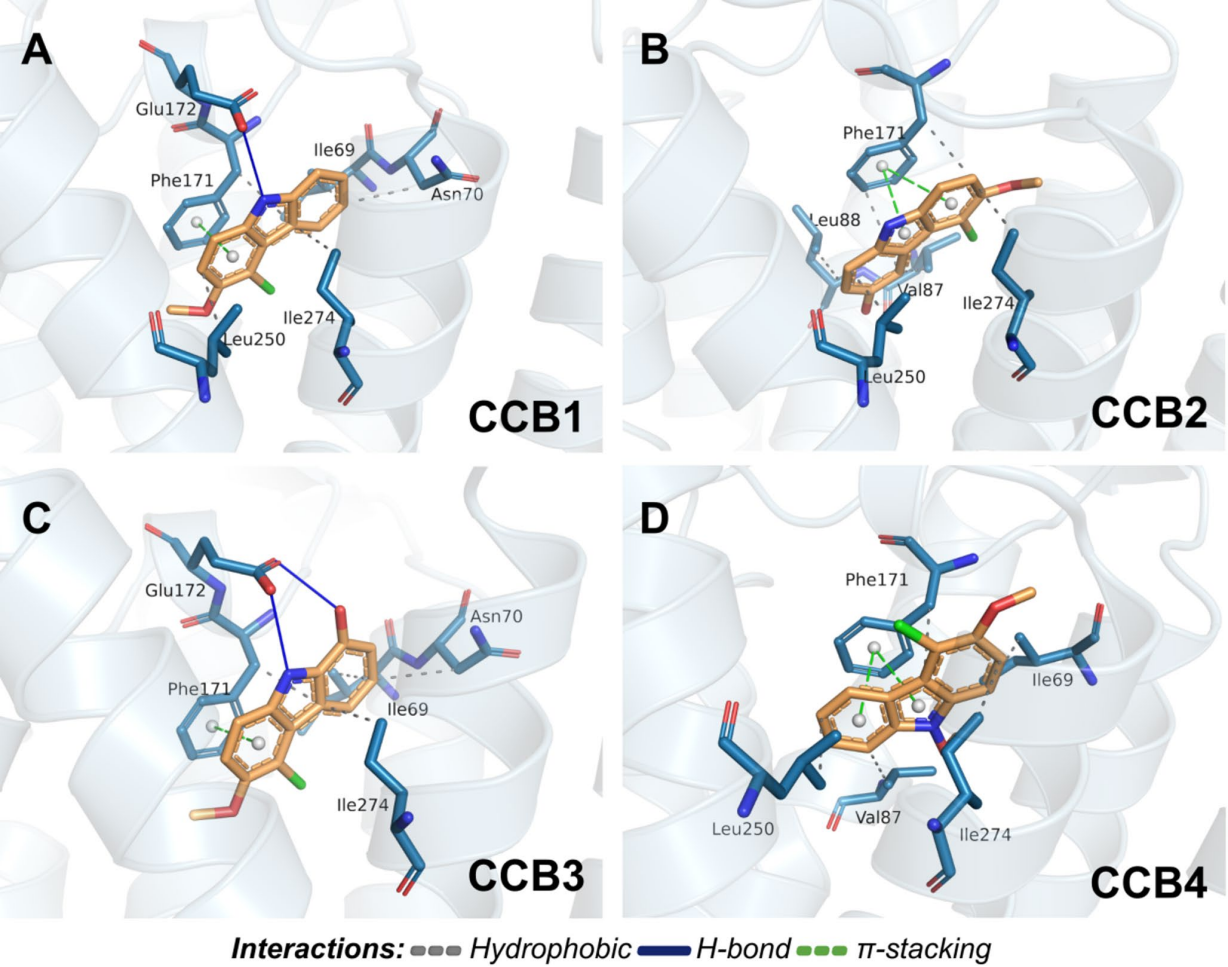
Structural contributions to ligand-receptor interactions between derivatives **A** CCB1, **B** CCB2, **C** CCB3, and **D** CCB4 and amino acid residues of the A_1_R receptor active site

The analysis of the structural contributions enabled the observation of the types of interactions formed by the CCB1-4 derivatives with the residues in the A_1_R binding site. It is noteworthy that the aromatic system involving the heteroaromatic ring containing the amine (–NH) and the chloro-substituted benzene contributed to the formation of π-stacking interactions with the aromatic portion of the CCB1-3 derivatives. A portion of the Phe171 residue (Fig. 6) exhibited a relative distance of 3.8–3.9 Å between the aromatic rings of the ligands and the aromatic ring of the residue (Table 1) [46]. It is noteworthy that in the CCB1 and CCB3 derivatives, the parallel stacking between the chloro-substituted aromatic systems and the Phe171 residue facilitated the formation of hydrogen interactions between the amine group (-NH) of the ligands and the carboxylate group (–COO^–^) of the Glu172 residue was also observed to interact with the heteroaromatic group, which increased the prevalence of hydrophobic interactions in compounds CCB2 and CCB4 (Fig. 6).

Additionally, it was observed that the CCB3 ligand possesses a substituted hydroxyl group (–OH) on the benzene ring fused to the heteroaromatic ring, which functions as a hydrogen donor for the carbonyl of the polar side chain of the Glu172 residue. (R–OH···O = C–R) [46], a donor-acceptor distance of approximately 2.09 Å, indicating that the compound can bind more strongly to the protein than other analogues [54].

**Table 1:** Data from molecular Docking simulations expressed in RMSD and affinity energy (*E*_A_), and details of ligand-receptor interactions expressed in type, residue, and distance

Ligand	RMSD	*EA*	Ligand-receptor interactions	
			Type	Residue (Distance)
CCB1	1.641 Å	− 8.263 kcal/mol	Hydrophobic	Ile69 (3.43 Å), Asn70 (3.55 Å), Phe171 (3.67 Å), Leu250 (3.94 Å), Ile274 (3.49 Å)
			H-bond	Glu172 (2.38 Å)
			π-stacking	Phe171 (3.94 Å)
CCB2	1.601 Å	− 8.5 kcal/mol	Hydrophobic	Val87 (3.42 Å), Leu88 (3.92 Å), Phe171 (3.52 Å), Phe171 (3.98 Å), Leu250 (3.72 Å), Ile274 (3.48 Å)
			π-stacking	Phe171 (4.09 Å), Phe171 (3.81 Å)
CCB3	1.591 Å	− 8.642 kcal/mol	Hydrophobic	Ile69 (3.54 Å), Asn70 (3.69 Å), Phe171 (3.55 Å), Ile274 (3.49 Å)
			H-bond	Glu172 (2.09 Å), Glu172 (2.27 Å)
			π-stacking	Phe171 (3.85 Å)
CCB4	1.518 Å	− 8.584 kcal/mol	Hydrophobic	Ile69 (3.64 Å), Val87 (3.60 Å), Phe171 (3.62 Å), Leu250 (3.74 Å), Ile274 (3.72 Å)
			π-stacking	Phe171 (3.90 Å), Phe171 (3.85 Å)
ADN*	1.098 Å	− 5.526 kcal/mol	H-bond	Glu172 (2.41 Å), Asn184 (3.02 Å), Asn254 (1.96 Å), Asn254 (2.11 Å), His278 (2.74 Å)
ASP5854*	1.649 Å	− 6.428 kcal/mol	Hydrophobic	Thr270 (3.67 Å), Tyr271 (3.46 Å)
			H-bond	Tyr271 (1.98 Å)

*Endogenous ligand used as a comparison in molecular docking simulations

### Molecular dynamics simulations

#### RMSD variations analysis

**Fig. 7:**
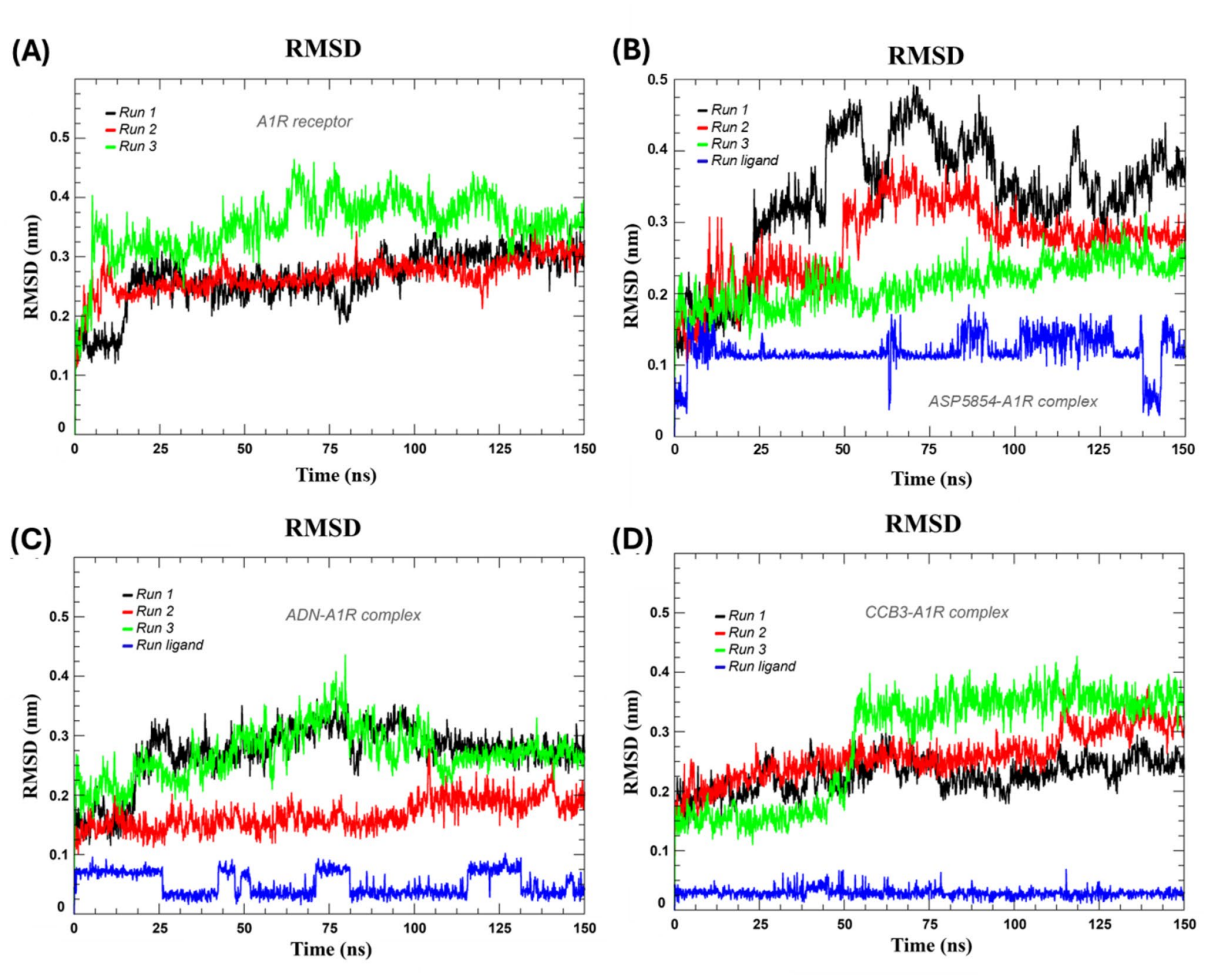
RMSD variations **A** System formed with the presence of the A_1_R receptor, **B** system formed by the antagonist ASP5854 **C** conformational variations with the co-crystallized inhibitor and **D** RMSD values for the system formed with the CCB3 ligand

Following the completion of the MD simulations, the proposed conformational values for each system were evaluated. Additionally, the potential variations across each system were observed through the analysis of RMSD (Fig. 7). The A_1_R receptor was initially subjected to a molecular dynamics simulation in the absence of non-protein ligands in its interaction cavities (Fig. 7A). This enabled the analysis of the protein’s intrinsic conformational variations. Figure 7B illustrates the variations observed between the receptor and the ADN agonist, while Fig. 7C depicts the RMSD values for the complex formed between the CCB3 ligand and the A_1_R receptor. The molecular dynamics simulations were performed in triplicate. The data from each replicate is represented by black (run 1), red (run 2), and green (run 3) lines in Fig. 12. Figure 12B, C, and D show the blue lines corresponding to the RMSD values of the ligands, illustrating the conformational changes to which each ligand was subjected along the trajectory.

When examining the RMSD variations of the receptor in Fig. 7A, it is evident that the system exhibited significant fluctuations from the outset of the simulation, with variations of 1.6 Å observed over a period of 5 ns in the first run. In the second run, the conformational variation was 2.4 Å, while the third simulation showed values close to 3.6 Å. The first and second simulations showed cohesive values from 15 ns, with results close to 2.5 Å, and remained relatively cohesive up to 150 ns, with conformational variations around 3.0 Å. On the other hand, when dealing with the third simulation, it was observed that the system reached a peak in variation with RMSD values around 4.5 Å and then exhibited more minor deviations, ultimately reaching a value of 3.1 Å at the end of the simulation.

Considering the MD studies associated with the biological receptor A_1_R, Fig. 7B demonstrates the RMSD values ​​associated with the system formed with the antagonist ASP5854. The data indicated that, from the beginning of the simulations until approximately 20 ns, variations of approximately 2.0 Å were detected for the three runs. After 25 ns, it was found that the three runs presented divergence indices with values close to 3.0 Å (run 1), 2.3 Å (run 2) and 2.0 Å (run 3). The highest variation index was associated with run 1, reaching a deformation equivalent to 4.9 Å (70 ns), while for run 2 the values were close to 3.2 Å and 2.1 Å (run 3). As the end of the 500 ns simulation approached, greater cohesion between the runs was observed, with values at the end of the simulation being 3.8 Å (run 1), 2.9 Å (run 2), and 2.6 Å (run 3). Regarding the variations indicated by the changes in the antagonist ASP5854 (run ligand), it was detected that at the beginning of the simulation up to 5 ns, the value referring to the ligand itself was around 0.5 Å. Subsequently, a significant increase was observed, reaching values around 1.2 Å, where it remained relatively constant until reaching 138 ns (0.5 Å). However, at the end, at 150 ns, the result showed a return to the 1.2 Å level, indicating an oscillation linked to the antagonist.

The MD simulations conducted with the A_1_R receptor in the presence of the endogenous ADN ligand (Fig. 7C) demonstrated two distinct behaviours in the evaluated runs. In the initial and third simulations, the systems demonstrated stabilization and cohesion, with deviations of 3.0 Å at 22 ns, reaching conformational variations of 3.5 Å at 75 ns for the first run and 4.0 Å for the third. Both reached a stable position at 2.8 Å by 150 ns. The second run demonstrated a lower degree of variation, with a value of 1.5 Å at three ns, remaining constant until 100 ns, and subsequently rising to 2.1 Å until 150 ns. About the RMSD of the ligand, there were four instances of instability: between 1 ns and 26 ns (0.7 Å), 40 ns and 51 ns (0.6 Å), 71 ns and 81 ns (0.8 Å), and 115 ns to 130 ns (0.7 Å). Subsequently, the system exhibited stability with an RMSD of 0.5 Å until the conclusion of the simulation at 150 ns.

In the system constituted by the CCB3-A_1_R complex (Fig. 7D), the initial two runs exhibited comparable RMSD values of approximately 2.5 Å up to 110 ns. From this point onwards, the first run remained stable, with variations of 2.8 Å until the end of the simulation. In contrast, the second run showed peaks of up to 3.0 Å at 150 ns. The third run initially exhibited fluctuations of 1.6 Å up to 50 ns, followed by an increase to 3.6 Å at 55 ns, and then remained stable up to 150 ns. About the CCB3 ligand, minimal fluctuations were observed throughout the simulation, with constant values of 0.35 Å.

The variation values observed in Fig. 7A indicates that the A_1_R receiver exhibited values that highlight potential instabilities, primarily associated with the third run. In contrast, the first and second runs demonstrated good initial values. However, as the simulation neared its end, the similarity observed between the first and second runs was disrupted, indicating that the system exhibited instability due to its RMSD variations and possibly increasing divergent RMSD variations [55].

During the analysis of the ASP5854-A_1_R system, it was found that the variation values ​​indicate low cohesion between runs. This may negatively impact the potential conformational stabilization expressed by the biological complex. Regarding the variations related to the ligand moiety (blue line), it is clear that the stabilization principles associated with cohesive variation are detected. However, at different points in the 500 ns simulations, the values showed more pronounced variation rates, exposing possible moments of instability caused by the conformational change undergone by the analyzed ligand.

An evaluation of the system formed with the inhibitor, characterised by the presence of the ADN-A_1_R complex, revealed that the system exhibited favourable stability indices, particularly during the second run. Another favourable point is related to the presence of cohesion between the first and second runs. The evaluation of the conformational variations of the inhibitor itself (blue) revealed low conformational variations, accompanied by minor structural variations. The ligand demonstrated favourable results about stability, exhibiting values of approximately 0.5 Å up to 150 ns. This suggests the potential for inhibition resulting from the involvement of the ADN inhibitor and its predominant association with RMSD variations [56].

Upon analyzing the results expressed by the presence of the CCB3 compound in direct complex with the A_1_R receptor, it can be observed that the system presented stability indices closely associated with the first and second runs, exhibiting low RMSD values. However, upon closer examination of the unique variations in the ligand, it can be seen that the compound demonstrates low RMSD variations and excellent cohesion and continuity up to 150 ns, indicating a preference for specificity about the cavity in which it is inserted. This may be a determining factor in highlighting the potential for inhibition associated directly with the CCB3 compound in complex formation with the A_1_R receptor [56, 57].

#### Variations of fluctuations by RMSF

**Fig. 8:**
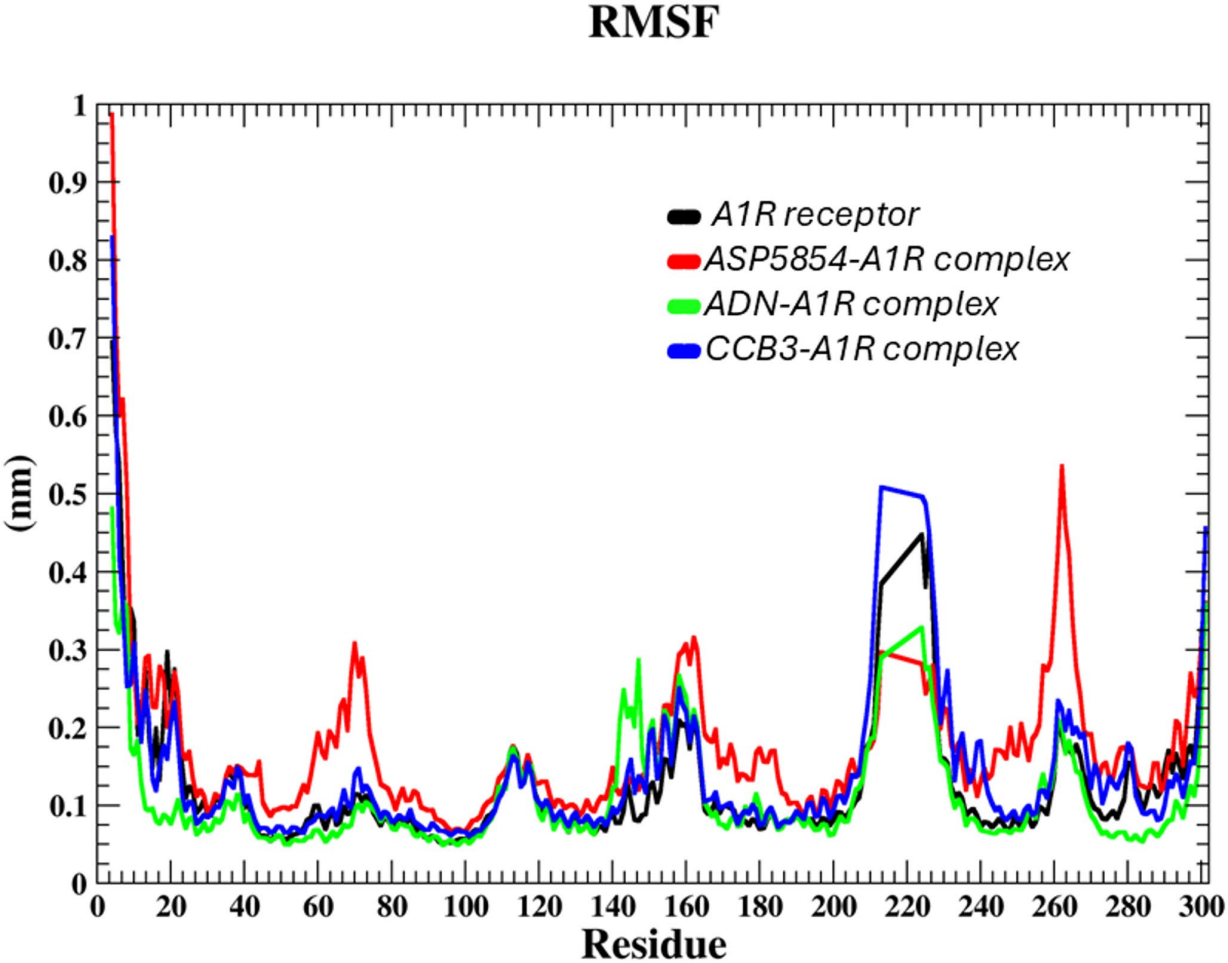
Fluctuations of amino acid residues of the target receptor (black line), the A_1_R receptor without the presence of ligand (red line), the system formed with the presence of the ADN-A_1_R complex (green line), and fluctuations of the CCB3-A_1_R complex

The variations in RMSF are directly associated with the potential rigidity or flexibility of some amino acid residues present in the target receptors. This suggests that deformations in amino acids may be identified by analysing the initial and final states of the MD simulation. The fluctuations observed in Fig. 8 were due to three different systems. The first was the A_1_R receptor in the absence of a ligand (black), the second was the complex formed, associated with the contribution of the antagonist ASP5854 (red). In contrast, the system constructed by the A_1_R receptor and the co-crystallized inhibitor ADN was shown in green, and the third RMSF variation was related to the CCB3-A_1_R complex (blue).

The amino acid residue fluctuations between the systems demonstrated a high degree of similarity, indicating that most residues exhibit similar flexibility. However, certain residues were observed to exhibit significant discrepancies. For example, the systems generally exhibited a range of significant variations, particularly in the ASP5854-A_1_R complex. The primary indices were observed between residues Leu65 and Thr75, with a value of approximately 3.1 Å. A more pronounced range of variation was evident between amino acids Ala158 and Phe185, with a value greater than 3.0 Å. The most substantial discrepancy reported in the ASP5854-A_1_R system was between amino acids Cys260 and Ile268, with a variation value of 5.4 Å. Specifically, the evaluation of residue Gly145 revealed fluctuation values of 1.0 Å in the A_1_R system, while for the antagonist, its value was equivalent to 1.5 Å, 2.3 Å in the ADN-A_1_R complex, and 1.6 Å in the CCB3-A_1_R complex. The A_1_R complex is the subject of this study. This divergence pattern persisted up to residue Ala158, which exhibited variations of 2.1 Å, 2.6 Å, and 2.5 Å, respectively, in the three systems. Another point of divergence was observed at residue 213, which exhibited fluctuations of 3.8 Å in A_1_R, while for the ASP5854-A_1_R complex a value of about 3.1 Å was observed, 3.0 Å for the ADN-A_1_R system and 5.1 Å in CCB3-A_1_R. This finding indicates substantial disparities in local flexibility. In comparison, the system formed by the ASP5854-A_1_R complex exhibited the highest observed fluctuation rates at distinct amino acid residues, directly corroborating the RMSD data presented. Conversely, the A_1_R, ADN-A_1_R, and CCB3-A_1_R systems exhibited smaller deformations, indicating reduced flexibility compared to the antagonist.

The RMSF variations indicate that the system formed only with the target receptor sometimes exhibited minor deformations in its amino acid residues. Still, when compared directly with the fluctuation variations of the ADN-A_1_R complex, it can be seen that this system showed more minor deformations in general, However, when observing the deformations expressed by the CCB3-A_1_R complex, it can be seen that there were similar deformations when compared to the complex formed with the inhibitor, corroborating the potential stability and inhibition potential of the A_1_R receptor [58].

#### Occupancy H-bond analysis

**Fig. 9:**
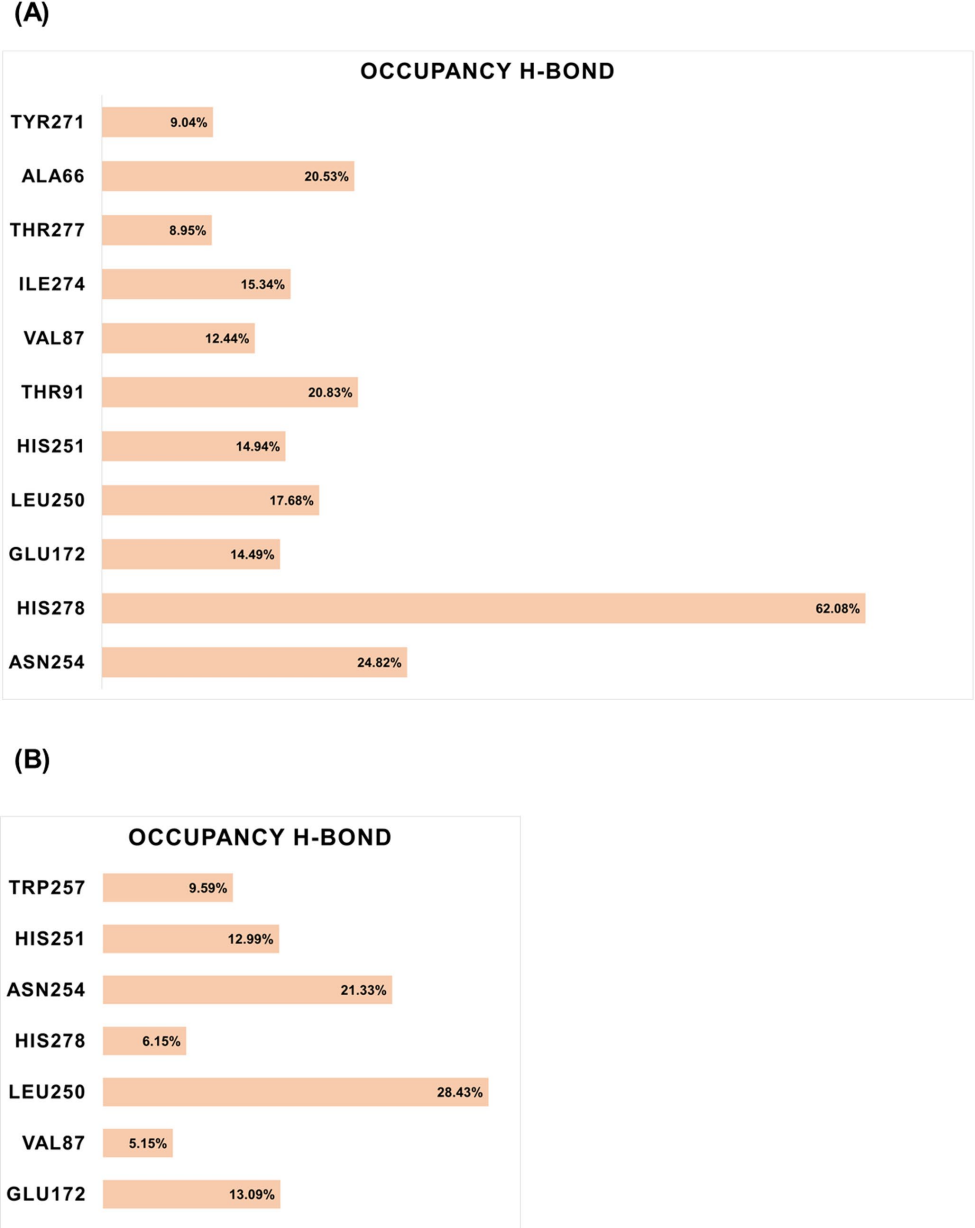
Occupancy of hydrogen bonds, **A** Hydrogen bonds formed with the compound ADN and A_1_R, **B** percentage of hydrogen bonds existing between the compound CCB3 and the receptor A_1_R

The formation of hydrogen bonds between the A_1_R receptor and the ligands ASP5854, ADN and CCB3, with the main most relevant variations, observed in Fig. 9. This figure depicts the occupancies of the hydrogen bonds formed during the MD simulations over a 150 ns period, with percentage values deemed significant when exceeding 5.0% [59].

Considering molecular dynamics studies, the system formed by the contribution of the ASP5854 ligand to the amino acid residues present in the interaction cavity is observed. After verifying the occupancy of the hydrogen bonds, it is indicated that only three relevant bonds were formed. These bonds are indicated by the amino acid residues Leu242, Gly279, and Leu238, with occupancy values ​​of 9.79%, 10.64%, and 8.14%, respectively.

When analysing the occupancy values of the hydrogen bonds formed between the endogenous ADN agonist and the residues of its binding site (Fig. 9A), the residues of Tyr271 (9.04%), Ala66 (20.53%), Thr277 (8.95%), Ile274 (15.34%), Val87 (12.44%), Thr91 (20.83%), His251 (14.94%), Leu250 (17.68%), Glu172 (14.49%), His278 (62.08%) and Asn254 (24.82%) stand out. In this relationship, it is worth noting that the residues Thr277, Ile274, Val87, Thr91, Leu250, Glu172, His278, and Asn254 comprise the active site of the A_1_R receptor. On the other hand, the complex formed between the CCB3 ligand and the A_1_R receptor (Fig. 9B) showed hydrogen bond occupancy with the residues of Trp257 (9.59%), His251 (12.99%), Asn254 (21.33%), His278 (6.15%), Leu250 (28.43%), Val87 (5.15%) and Glu172 (13.09%). In this list, only the His251 residue is not listed among the residues of the A_1_R receptor’s active site, indicating that the CCB3 compound forms a series of hydrogen bonds involving the polar portion of these amino acid residues.

Hydrogen bond occupancy analysis demonstrates that ASP5854 makes few contributions to the amino acid residues inserted into the interaction cavity, while the co-crystallized inhibitor DNA, present in the inhibition site, forms multiple hydrogen bonds directly with active site residues, exhibiting projections favourable to bond formation, which are beneficial for the interaction potential. Consequently, this increases the inhibition potential directly associated with the relationship between the ligand and the target receptor [60]. The analysis of the hydrogen bonds observed by the occupation revealed that the compound CCB3 formed hydrogen bonds with six amino acid residues present in the active site of the target receptor, indicating a high percentage of bonding. The compound CCB3 demonstrated stability at the inhibition site, with 28.43% of the interactions occurring through the Leu250 residue. This indicates a high potential for inhibiting the biological activity of the A_1_R target [61, 62].

#### MM/GBSA and free binding energy calculation

MD simulations demonstrated the stability behavior associated with the interaction between the ligand and the target receptor. Consequently, Table 2 presents the calculated values for all systems formed in the presence of ASP5854-A_1_R, ADN-A_1_R, and CCB3-A_1_R complexes, as determined by Molecular Mechanics/Generalized Born Surface Area (MM/GBSA) calculations. The free energy values for each complex were equal to -17.22 ± 4.56 kcal/mol (ASP5854-A_1_R), -23.32 ± 4.42 kcal/mol (ADN-A_1_R), and − 25.93 ± 4.13 kcal/mol (CCB3-A_1_R).

**Table 2:** Values referring to MM/GBSA, for the ASP5854-A_1_R (antagonist), ADN-A_1_R (agonist) and CCB3-A_1_R systems

Ligand-receptor complex	E_vdW_	E_ele_	G_GB_	G_SA_	− TΔS	ΔG_bind_
ASP5854-A_1_R	− 28.30	− 33.04	43.22	− 4.44	5.34	− 17.22 ± 4.56
ADN-A_1_R	− 29.76	− 36.01	41.86	− 4.68	5.27	− 23.32 ± 4.42
CCB3-A_1_**R**	− 29.88	− 35.89	40.20	− 5.54	5.18	− 25.93 ± 4.13

Molecular dynamics studies present a comprehensive overview of all contributions made in determining the binding free energy value for the ASP5854-A_1_R complex, as determined by the complexes formed between the ligands associated with the biological receptor. The data demonstrated that the system consolidated three favorable contributions to the binding free energy index formed by the terms E_vdW_ (-28.30 kcal/mol), E_ele_ (-33.04 kcal/mol), and G_SA_ (-4.44 kcal/mol), while only two unfavorable projections were observed: G_GB_ (43.22 kcal/mol) and -TΔS (5.34 kcal/mol). The integration of all these terms resulted in a good binding free energy value equal to -17.22 kcal/mol.

Table 2 presents the complete set of values associated with the MM/GBSA calculation, with a particular focus on the contributions made by each system. It is noteworthy that the ADN-A_1_R complex exhibited a free energy value of−23.32 kcal/mol, which was attributed to three favourable contributions to the free energy term. These contributions, namely van der Waals interaction energy (*E*_vdW_ =−29.76 kcal/mol), electrostatic energy (*E*_ele_ =−36.01 kcal/mol), and non-polar solvation energy by surface area (*G*_SA_ =−4.68 kcal/mol), collectively contributed to the overall free energy value observed for the complex. However, in the case of unfavourable contributions related to free energy, the terms polar solvation energy of the Generalized Born model (*G*_GB_ = 41.86 kcal/mol) and entropic correction (-TΔS = 5.27 kcal/mol) are highlighted. Upon evaluating the energy behavior, it was observed that three determining terms for the free energy were predominant, distributed among the *E*_vdW_, *E*_ele,_ and *G*_GB_ terms. The ligand-receptor relationship yielded an excellent free energy value, which corroborates the affinity of the complex [63]. The DM simulations yielded the MM/GBSA values for the CCB3-A_1_R complex, revealing a free energy value of−25.93 kcal/mol. This value was duly related to the calculated terms, which were as follows: It is possible to highlight three favourable contributions to the free energy, which are represented by the *E*_vdW_, *E*_ele,_ and *G*_SA_ terms, with values of−29.88,−35.89, and−5.54 kcal/mol, respectively. Regarding the unfavourable terms, it is possible to highlight the participation of the GGB (40.20 kcal/mol) and -TΔS (5.18 kcal/mol) terms. These results suggest that the *E*_vdW_, *E*_ele,_ and *G*_GB_ terms accounted for a significant proportion of the determinant contributions to the free energy potential [63, 64].

From the results presented, both complexes showed excellent free energy values. However, when comparing the two systems, it is worth emphasizing that the CCB3-A_1_R complex showed a better free energy order concerning the endogenous ADN ligand, thus demonstrating a high potential for stability and affinity, as observed in the DM simulations.

#### Conformational variation of complexes

**Fig. 10:**
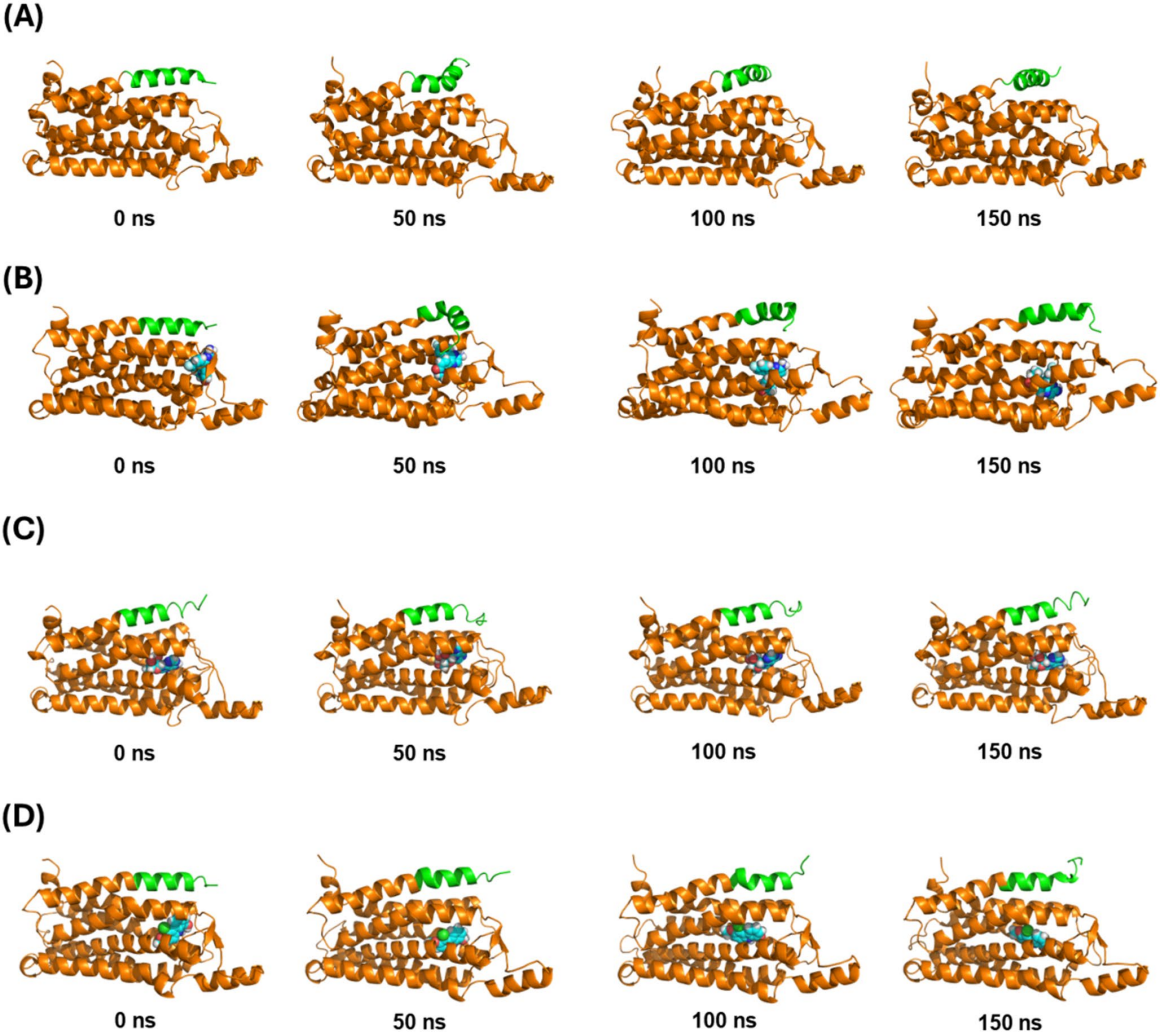
Visualization of system variations by MD: **A** system formed with the presence of the A_1_R receptor, **B** ASP5854-A_1_R complex **C** system for the ADN-A_1_R complex, **D** CCB3-A_1_R complex

About the structural variations of the systems under examination, it is possible to identify all the changes that occurred in specific frames of the simulations, specifically those involving the A_1_R receptor and the complexes formed with the ASP5854-A_1_R, ADN-A_1_R and CCB3-A_1_R systems (Fig. 10). This demonstrates how each system is composed and whether or not it is capable of incorporating a ligand into its coordinates. In these analyses, the structural changes in all the simulated systems were evaluated at specific time intervals: 0 ns (initial conformation), 50 ns, 100 ns, and finally 150 ns (final conformation). This allowed for an examination of how each system behaved during the DM simulations.

Upon examining the visualization of the MD prediction calculations, it can be observed that the system comprising solely the A_1_R receptor, as illustrated in Fig. 10A, exhibits minimal fluctuations in its amino acid residues between 0 ns and 150 ns. However, upon closer examination of the variations across different regions of the receptor, it becomes evident that it is possible to highlight the presence and movement of a loop (green), which shows peaks of instability as early as 50 ns. This is characterised by a deformation in its residues that move away from the other amino acid residues, remaining practically unchanged at 100 ns until the end of the simulation. This demonstrates the presence of a region in the receptor that exerts moments of instability.

The molecular dynamics studies revealed the primary conformational variations associated with the ASP5854-A_1_R complex, as depicted in Fig. 10B. The loop (green) undergoes substantial deformations between 50 and 100 nanoseconds, closely mirroring the behaviour of the isolated biological receptor. At 150 ns, it is observed that the loop presents a conformation similar to the initial one. The available data suggest that the ASP5854 compound may present a potential unstable effect in the initial portions of the simulation.

When the simulations were conducted with the presence of the co-crystallized ligand ADN (blue), as shown in Fig. 10C, it is initially noteworthy that the ligand exhibited excellent stability indices within the cavity where it is situated, demonstrating low structural variation within the system. When estimating the changes that occurred in the receptor containing ADN, it is highlighted that the loop (green), which underwent structural deformations when the receptor was alone, displayed a greater degree of stability from the onset of the simulation until 150 ns. This suggests that the presence of the nucleoside likely enhanced the stability of the A_1_R receptor, indicating an increased potential for inhibition with the presence of ADN.

Regarding the CCB3-A_1_R complex, as illustrated in Fig. 10D, the compound CCB3 (blue) demonstrated excellent stability within the system, with minimal structural variations over 150 ns, emphasizing an increase in its affinity. In terms of the analysis of structural modifications of the receptor, the loop (green) exhibited few changes throughout the simulation, indicating that the amino acid residues experienced minimal deformations. This evidence highlights stability points that directly correlate with the inhibition potential of the A_1_R receptor through its interaction with the ligand CCB3 [59].

#### PCA analysis

**Fig. 11:**
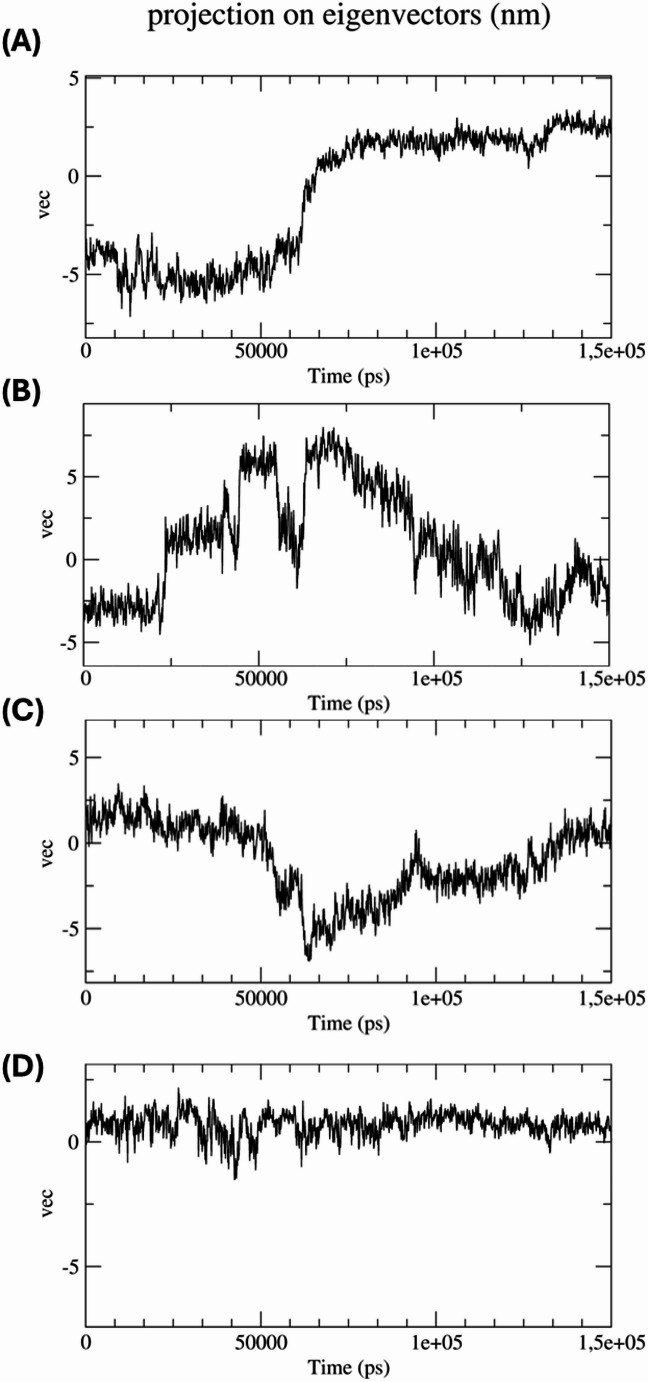
PCA analysis of molecular dynamics systems: **A** A1R receptor, **B** ASP5854-A_1_R complex **C** system for the ADN-A_1_R complex, **D** CCB3-A_1_R complex

Principal component analysis (PCA) studies, considering the projection onto the first eigenvector (vec), provided evidence indicative of significant differences in the protein’s conformational changes under different conditions. As illustrated in Fig. 11A, a conformational transition was observed at approximately 50 ns, with a shift from a negative projection to a positive one. This result demonstrates that the system explores diverse conformational states. Conversely, the presence of the antagonist (Fig. 11B) resulted in greater variations and jumps in conformational states, thus suggesting that binding possibly increases the protein’s overall flexibility, potentially resulting in structural instability.

Regarding the complexes with other ligands, relatively distinct profiles were revealed. The compound ADN (Fig. 11C) in complex with the receptor may be associated with a potential partial stabilization of the system, thus maintaining the biological receptor in a specific state before allowing new transitions, thus indicating a possible intermediate stability. Concomitantly, the ligand CCB3 (Fig. 11D) exhibited a more pronounced restriction of movement relative to the biological receptor, culminating in oscillations that remained close to zero throughout the simulation (150 ns). This finding suggests a potential significant stabilization of the structure. Taken together, these data reinforce the conclusion that the use of different compounds associated with the biological receptor can modulate movement vectors differently, directly interfering with conformational flexibility.

### MPO-based DMPK prediction

#### MLP analysis and druglikeness

In the research on the discovery of active drug compounds in the CNS, the incidence of apolar compounds leads to a series of potentially toxic analogs [65]. According to Wager et al. [12], acidic compounds that are poorly lipophilic at physiological pH (logD at pH 7.4 < 4.0) and larger and more polar than commonly active CNS compounds, with Topological Polar Surface Area (TPSA) between 40 and 90 Å^2^, exhibit a dynamic balance between high passive cellular permeability (*P*_app, A→B_ > 10 × 10 ^− 6^ cm/s) and low hepatic clearance (*CL*_int, u_ < 8.0 mL/min/kg) [13]. This multiparameter optimization (MPO) system ensures the selection of safe and pharmacologically viable compounds for new CNS therapies.

**Fig. 12:**
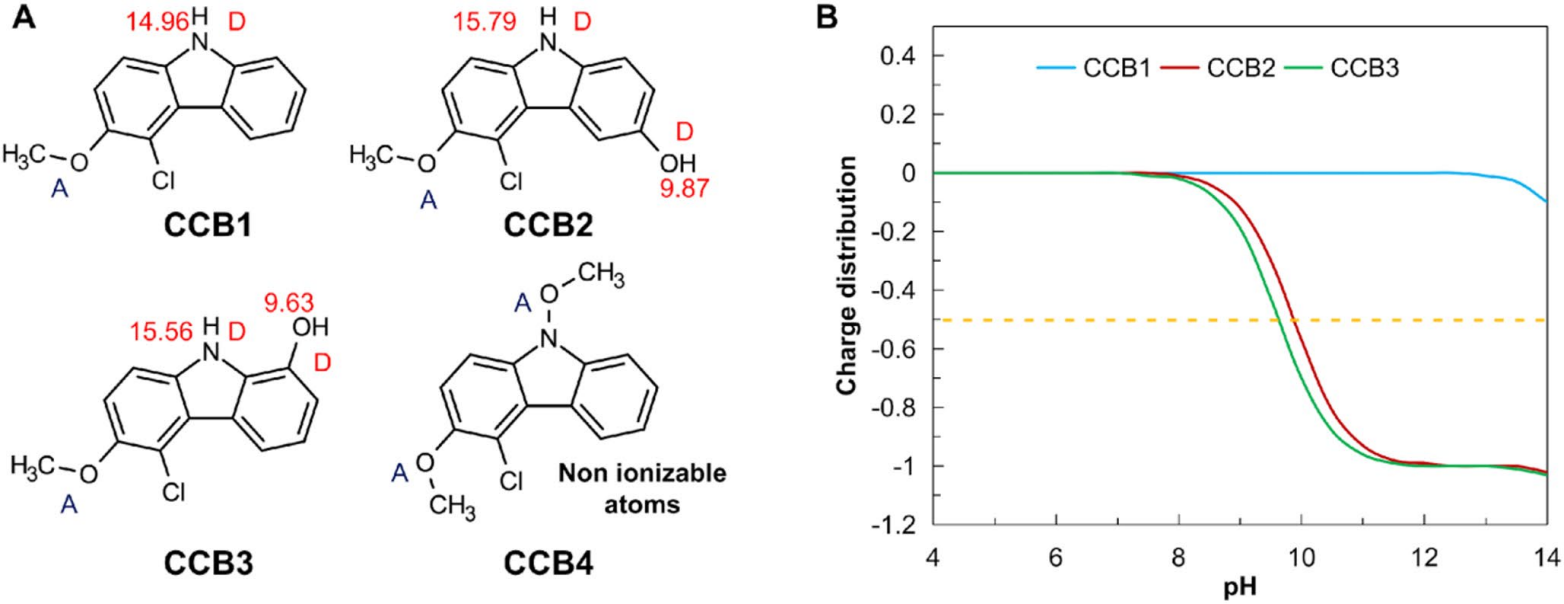
Acid-base analyses of the CCB1−4 derivatives are expressed in: **A** predicted pKa values concerning the hydrogen bond acceptor and donor groups, represented by the letters A and D, respectively, and **B** the variation of net charge as a function of pH changes

In an analysis of the acid-base equilibrium of compound derivatives, it was observed that hydroxylation at the C ring of the CCB2 and CCB3 analogs resulted in the formation of an acidic hydrogen bond donor center with pKa values of approximately 9.8 and 9.6, respectively (Fig. 12A). This indicates that the chemical equilibrium shifts toward the formation of the conjugate base with a net charge of−1 at basic pH levels. However, the acidic species predominates at pH 7.4 (Fig. 12B). The biological implications of this result are that neutral compounds cross the BBB more easily and can be better distributed to the CNS due to high oral bioavailability [66, 67]. In contrast, the methoxylation of the amino group in the carbazole skeleton leads to a chemical species devoid of ionizable atoms (Fig. 12A).

**Fig. 13:**
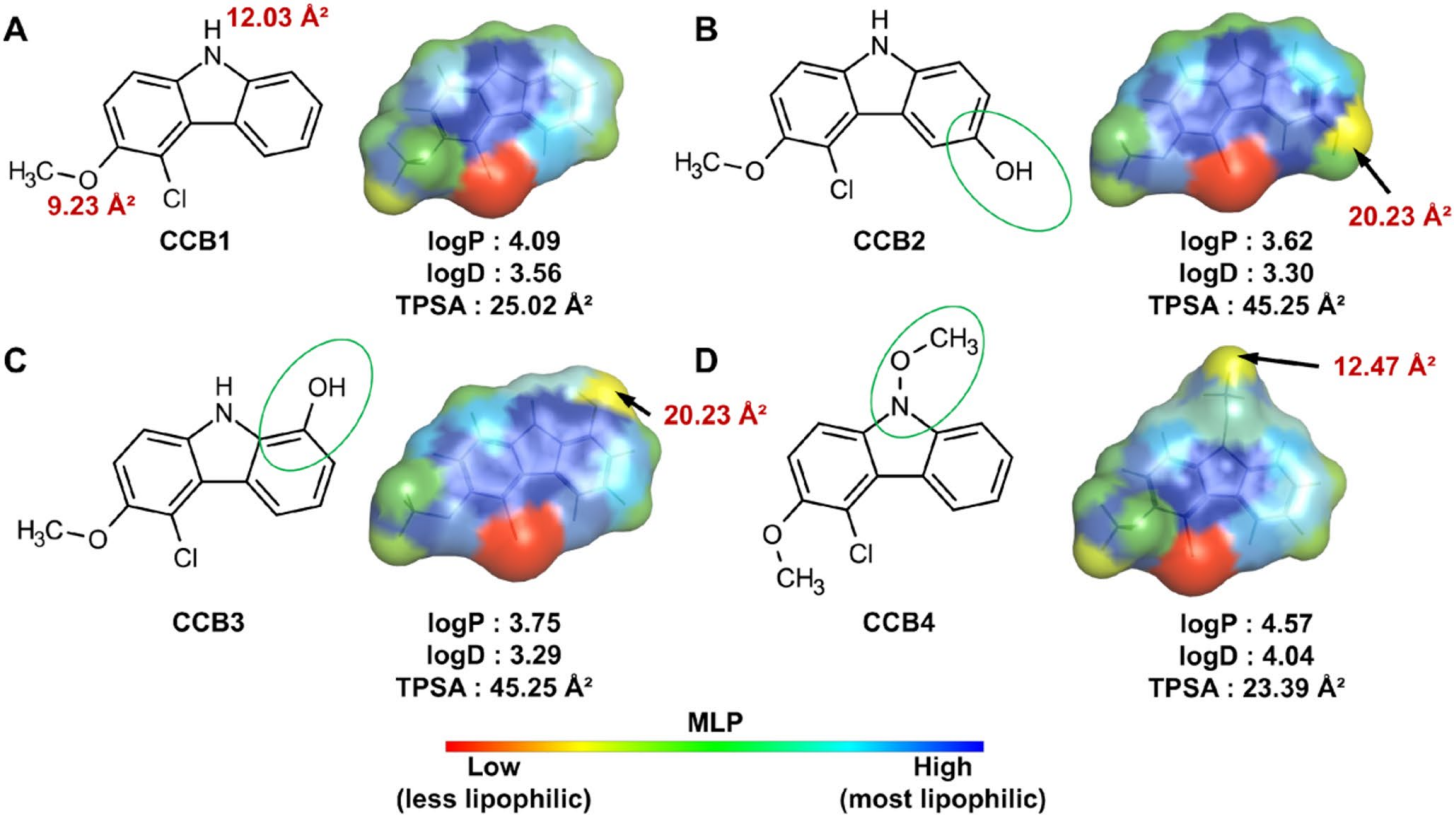
Surface map of molecular lipophilicity potential (MLP) and topological polar surface area (TPSA) for CCB derivatives, where the color spectrum varies from red, for the less lipophilic fragments, to blue, for the most lipophilic fragments: **A** CCB1, **B** CCB2, **C** CCB3 and **D** CCB4

In an analysis of molecular lipophilicity potential (MLP), the influence of the substituent groups of the CCB1−4 derivatives on their lipophilicity and topological polarity indices can be observed (Fig. 13) [36, 68]. The structures of CCB1-4 are predominantly hydrophobic in their aromatic rings, which may favor the formation of weak hydrophobic interactions with neuroreceptors (blue color spectra). It is noteworthy that the –OH group exhibits additive polarity properties (TPSA_OH_: 20.23 Å^2^) in the CCB2 and CCB3 compounds, thereby optimizing the TPSA to a threshold that favors BBB permeability with safety to the CNS, as per the established rule by Pfizer, Inc. (TPSA > 40 Å^2^) [12, 69]. This reduced logP to values closer to 3.0 for higher values, indicating a better alignment between lipophilicity and polarity (Table 3).

These topological analyses suggest that the CCB2−3 derivatives exhibit drug-like characteristics that align well with new structural trends for drugs acting in the CNS, demonstrating a low incidence of toxicity and optimized DMPK properties, with CNS MPO scores greater than 4.0 (Table 3) on a scale ranging from 0 to 6, reflecting pharmacokinetic viability [12, 14].

**Table 3:** Physicochemical properties calculated for CCB1−4 derivatives and applied to pfizer’s CNS MPO druglikeness criteria

Property	CCB1	CCB2	CCB3	CCB4
logP	4.09	3.62	3.75	4.57
logD at pH 7.4	3.56	3.30	3.29	4.04
TPSA	25.02 Å^2^	45.25 Å^2^	45.25 Å^2^	23.39 Å^2^
MW	231.05 g/mol	247.04 g/mol	247.04 g/mol	261.06 g/mol
HBD	1	2	2	0
pKa (basic)	4.87	5.51	4.59	2.99
CNS MPO score	3.76	4.54	4.48	3.38
Pfizer’s druglikeness rule*	Rejected	Accepted	Accepted	Rejected

*The druglikeness rule considers compounds that showed low lipophilicity at physiological pH (logD at pH 7.4 < 4) within the polarity range of low toxicity and pharmacokinetic viability (TPSA 40–90 Å^2^).

### Pharmacokinetic descriptors

The prediction of Parallel Artificial Membrane Permeation Assay (PAMPA) properties was conducted to estimate the potential cellular permeability of the compounds in cell lines commonly associated with human intestinal absorption (HIA) and BBB permeability [70, 71]. According to Pettersson et al., (2016) [13], well-absorbed compounds with safe CNS activity fall within the physicochemical space formed by substances exhibiting high cellular permeability in Madin-Darby Canine Kidney (MDCK) cells, with *P*_app, A→B_ > 5.0 × 10 ^− 6^ cm/s, and low intrinsic hepatic clearance, with *CL*_int, u_ < 8.0 mL/min/kg, as they demonstrate optimal Drug Metabolism and Pharmacokinetics (DMPK) efficiency.

**Fig. 14:**
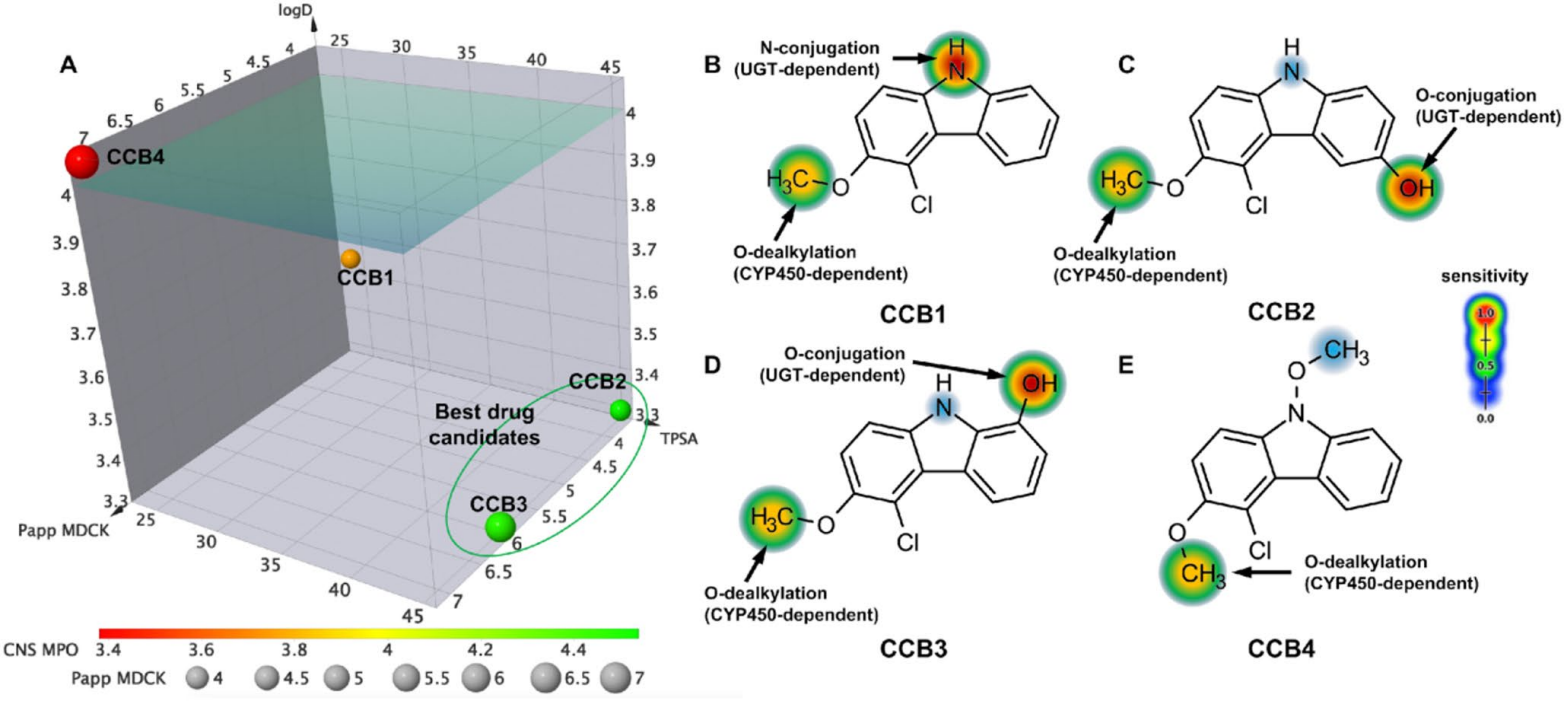
Graphical analyses of multiparametric optimization (MPO) and site of metabolism prediction (SOMP) of CCB1−4 derivatives: **A** alignment between buffer lipophilicity at physiological pH (logD) and topological polar surface (TPSA) in estimating passive cell permeability (*P*_app, A→B_) MDCK, considering CNS MPO scores. Site of metabolism sensitivity map of compounds **B** CCB1, **C** CCB2, **D** CCB3, and **E** CCB4

In an analysis of the MPO system, depicted in Fig. 14A, it is evident that the compound CCB4 exhibits high buffer lipophilicity at physiological pH, with a logD at pH 7.4 > 4.0, resulting in a CNS MPO score of approximately 3.38. Compounds within this lipophilicity range can be metabolically unstable and potentially toxic to the CNS [65, 72]. Within the acceptable lipophilicity range, it was observed that compounds CCB2 and CCB3 display a TPSA that ensures greater CNS safety, with a calculated polar surface area of 45.25 Å^2^ (Fig. 14A), resulting in CNS MPO scores of approximately 4.54 and 4.48, respectively (Table 3). These results suggest that the CCB2-3 derivatives exhibit optimal pharmacokinetics in terms of permeability and metabolic stability.

An analysis of the pharmacokinetic descriptors revealed that *P*_app, A→B_ Caco-2 in the order of 10^− 5^ cm/s indicates that the compounds exhibit high permeability in the intestinal epithelium, thereby substantiating HIA > 92% (Table 3) [73, 74]. For *P*_app, A→B_ MDCK, predicted values in the order of 10^− 5^ cm/s are indicative of high permeability in BBB-like membranes. The values are moderate for compounds with moderate lipophilicity (logP around 3.0), i.e., CCB2-3, with BBB distribution in the order of ~ 6.9 (*C*_brain_/*C*_blood_) [71]. The combination of these values with low clearance (*CL*_int, u_ < 5.6 mL/min/kg) and low probability of being a P-gp substrate predicts good oral bioavailability in blood plasma [13]. In conjunction with a PPB of less than 50%, a substantial free molecular fraction of CCB1-3 compounds is anticipated to be distributed to biological tissues, including the BBB [67, 75] (Table 4).

**Table 4:** Pharmacokinetic properties of CCB1-4 derivatives expressed as PAMPA descriptors, CNS permeability, and metabolic stability

Property	CCB1	CCB2	CCB3	CCB4
*P*_app, A→B_ Caco−2	1.77 × 10 ^− 5^ cm/s	1.51 × 10 ^− 5^ cm/s	2.08 × 10 ^− 5^ cm/s	2.34 × 10 ^− 5^ cm/s
*P*_app, A→B_ MDCK	3.80 × 10 ^− 5^ cm/s	3.66 × 10 ^− 5^ cm/s	6.02 × 10 ^− 5^ cm/s	7.14 × 10 ^− 5^ cm/s
*CL*_int, u_	4.49 mL/min/kg	4.77 mL/min/kg	5.59 mL/min/kg	5.23 mL/min/kg
P-gp substrate	0.21	0.14	0.14	0.25
HIA	95.61%	92.39%	92.39%	~ 100%
PPB	47.99%	49.63%	42.17%	56.08%
logBB	−1.57	−1.56	−1.39	−1.46
BBB	11.06	6.91	6.91	1.83
CYP2C9 substrate	0.99	0.97	0.98	0.94
CYP2D6 substrate	0.70	0.65	0.65	0.70
CYP3A4 substrate	0.92	0.77	0.68	0.79

The machine learniSng model employed in this predictive study assesses the probability that CCB1-4 derivatives will impact various organ toxicity endpoints, encompassing physiological systems such as the blood, heart, digestive system, liver, and skin. This model determines whether the compounds follow a structural trend within the recommended therapeutic range, providing greater precision in the predictions. The module used provides percentage estimates for each organ system, including the nervous, auditory, hepatic, renal, and cardiovascular systems. These predictions are based on a training set designed to recognize reactive molecular fragments, enabling structural similarity testing to anticipate a range of adverse effects in potential drug candidates, thereby ensuring a more robust and informed risk assessment [38, 76].

**Fig. 15:**
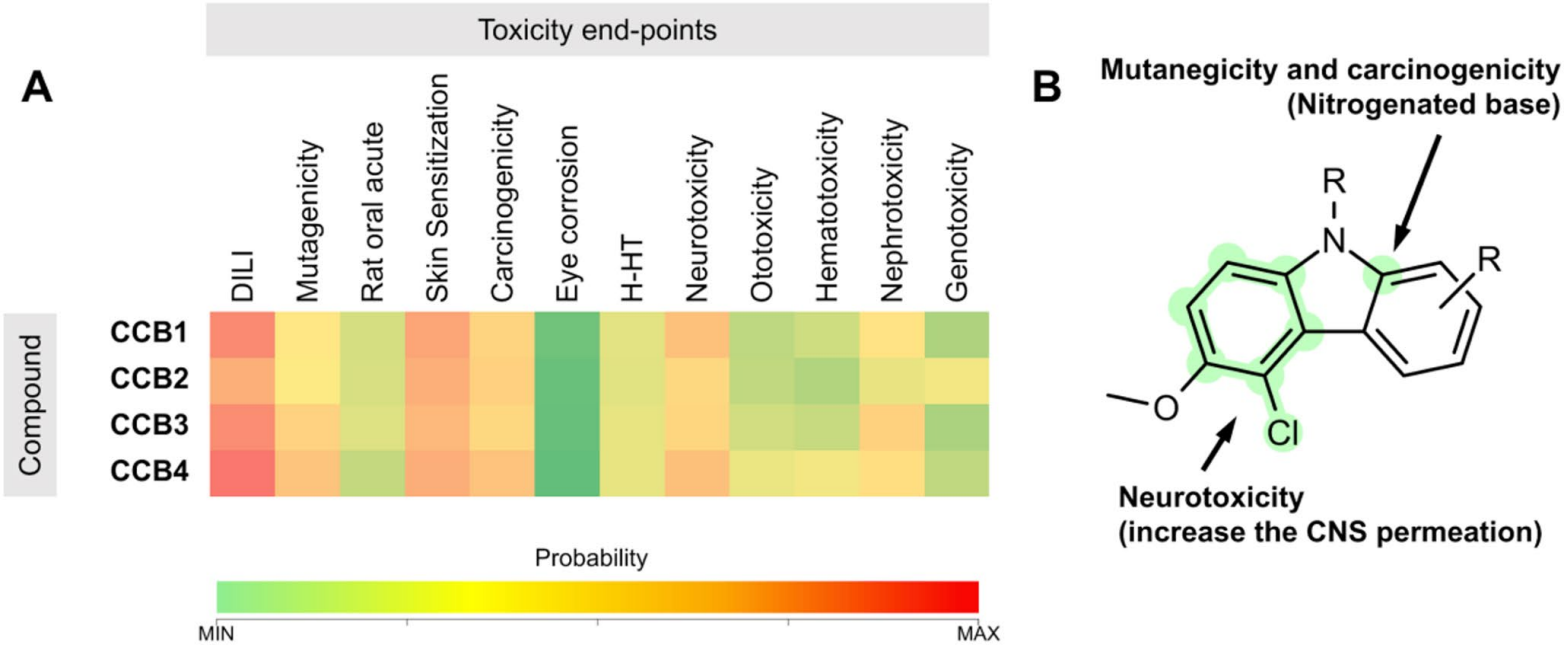
Results of toxicity spectrum prediction expressed in: **A** end-point toxicity heatmap, where the color spectrum varies from green (0.0) to red (0.95), and **B** prediction of toxicophores in the ADMETlab 3.0 server

The probability values predicted by the similarity test with reactive compounds are shown in Table 5. When analyzing the contributions of toxicity descriptors, there is a clear tendency for compounds to induce liver damage (DILI) and neurotoxic responses. This finding may be consistent with the probability that the compounds will be metabolized by CYP2C9, 2D6, and 3A4 in the human liver (Fig. 14B–E), resulting in the formation of reactive species that may be distributed to the CNS (Fig. 15A). In the identification of interfering structures, the aromatic ring with –Cl and –OCH_3_ substitutions has been associated with this neurotoxic risk. Specifically, –Cl has been demonstrated to increase the lipophilicity of the compound and its permeability, while –OCH_3_ can be metabolized in the hepatic microsomal system (Fig. 15B) [77, 78].

**Table 5:** Predicted organ toxicity descriptors for CCB1-4 derivatives using admetlab 3.0 predictor

Comp	Toxicity end-points											
	DILI	Muta	ROA	Skin	Carcino	EC	H-HT	Neuro	Oto	Hemato	Nephro	Geno
CCB1	0.91	0.71	0.51	0.85	0.75	0.06	0.58	0.79	0.41	0.48	0.72	0.34
CCB2	0.83	0.69	0.52	0.83	0.76	0.03	0.57	0.74	0.42	0.35	0.61	0.64
CCB3	0.90	0.76	0.56	0.81	0.74	0.02	0.59	0.75	0.50	0.45	0.76	0.32
CCB4	0.95	0.78	0.43	0.83	0.79	0.00	0.60	0.79	0.61	0.65	0.73	0.42

DILI: Drug-Induced Liver Injury; Muta: Mutagenicity; ROA: Rat Oral Acute toxicity; Carcino: Carcinogenicity; EC: Eye corrosion; H-HT: Human Hepatotoxicity; Neuro: Neurotoxicity; Oto: Ototoxicity; Hemato: Hematotoxicity; Nephro: Nephrotoxicity; Geno: Genotoxicity

## Conclusion

In this study, a ligand-based virtual screening approach demonstrated that the chlocarbazomycins scaffold is a key structure in the discovery of new A_1_R modulators. In accordance with these findings, molecular docking simulations revealed that they can bind to the orthosteric site of A_1_R, forming polar interactions analogous to the endogenous substrate ADN, by interacting with the Glu172 residue. Furthermore, these interactions suggest that they compete for the same binding site as the antagonist ASP5854 and act synergistically with the allosteric modulator MIPS521. DFT calculations suggest that CCB3 and CCB4 better accommodate the extra charge received in intermolecular interactions.

Molecular dynamics simulations estimated a free binding energy of − 25.93 kcal/mol for the CCB3 derivative, which obtained the highest negative affinity energy when interacting with A_1_R. Furthermore, pharmacokinetic parameters indicate that these molecules exhibit significant permeability in MDCK BBB-like cells, suggesting a potential for CNS targeting. However, the pharmacologically active principle is theoretically based on controlling the daily oral dose administered.

## Supplementary Information

Below is the link to the electronic supplementary material.

Supplementary Material 1

## Data Availability

No datasets were generated or analysed during the current study.
