# Supplementary material for: Natural chlocarbazomycins as potential adenosine A1 receptor antagonists: ligand-based and structure-based virtual screening, quantum chemical analysis and CNS MPO study
Source: Mol Divers. 2026 Jan 14;30(4):5593–619. doi: 10.1007/s11030-025-11446-6 (PMC13332976; doi:10.1007/s11030-025-11446-6)

**Natural Chlocarbazomycins as Potential Adenosine A1 Receptor Antagonists: Ligand-Based and Structure-Based Virtual Screening, Quantum Chemical Analysis and CNS MPO Study**

**Supplementary data**

$\Delta E_{gap}=E_{LUMO}-E_{HOMO}$ Eq. 1

$IE=-E_{HOMO}$ Eq. 2

$EA=-E_{LUMO}$ Eq. 3

$\chi=\left( \frac{\partial E}{\partial N} \right)_{v_{\left( r \right)}}$ Eq. 4

$\mu=-\chi$ Eq. 5

$\eta=\frac{1}{2}\left( \frac{\partial\mu}{\partial N} \right)_{v_{\left( r \right)}}=\frac{1}{2}\left( \frac{\partial^{2}E}{\partial N^{2}} \right)_{v_{\left( r \right)}}$ Eq. 6

$\chi=-\mu=\frac{1}{2}\left( IE+EA \right)=\frac{-1}{2}\left( E_{HOMO}+E_{LUMO} \right)$ Eq. 7

$\eta=\frac{1}{2}\left( IE-EA \right)=\frac{-1}{2}\left( E_{HOMO}-E_{LUMO} \right)$ Eq. 8

$S=\frac{1}{\eta}$ Eq. 9

$\omega=\frac{\mu^{2}}{2\eta}$ Eq. 10

$\varepsilon=\frac{1}{\omega}$ Eq. 11

$f\left( r \right)=\left[ \frac{\partial\rho_{\left( r \right)}}{\partial N} \right]_{v_{\left( r \right)}}$ Eq. 12

$f_{A}^{+}=q_{N}^{A}-q_{N+1}^{A}$ Eq.13

$f_{A}^{-}=q_{N-1}^{A}-q_{N}^{A}$ Eq.14

$f_{A}^{0}=\frac{\left( q_{N-1}^{A}-q_{N+1}^{A} \right)}{2}$ Eq.15

$\Delta f=f_{A}^{+-f_{A}^{-}}$ Eq.16

$\Delta\omega=\omega\Delta f$ Eq.17

Supplementary Table S1**.** Calculated chemical descriptors for CCB1–4 in chloroform, DMSO, methanol, and water using the B3LYP/6-311++G(d,p) method.

| **CCB1** | | | | | |
| --- | --- | --- | --- | --- | --- |
| **Descriptos** | **Chloroform** | **DMSO** | **Methanol** | **Water** |  |
| **HOMO energy** $E_{HOMO}$**(eV)** | -5.579 | -5.626 | -5.626 | -5.629 |  |
| **LUMO energy** $E_{LUMO}$**(eV)** | -1.292 | -1.344 | -1.341 | -1.347 |  |
| **Gap energy (**$\Delta E_{GAP},$**eV)** | 4.287 | 4.282 | 4.285 | 4.282 |  |
| **Ionization Energy (**$IE$**, eV)** | 5.579 | 5.626 | 5.626 | 5.629 |  |
| **Electronic Affinity (**$EA,$**eV)** | 1.292 | 1.344 | 1.341 | 1.347 |  |
| **Eletronegativity (**$\chi,$**eV)** | 3.436 | 3.485 | 3.484 | 3.488 |  |
| **Global Chemical Hardness (**$\eta,$**eV)** | 2.144 | 2.141 | 2.143 | 2.141 |  |
| **Global Chemical Softness (**$S,$ **eV^-1^)** | 0.467 | 0.467 | 0.467 | 0.467 |  |
| **Global Electrophilicity Index (**$\omega,$**eV)** | 2.753 | 2.836 | 2.832 | 2.841 |  |
| **Global Nucleophilicity Index (**$\epsilon,$**eV^-1^)** | 0.363 | 0.353 | 0.353 | 0.352 |  |
| **CCB2** | | | | | |
| **Descriptos** | **Chloroform** | **DMSO** | **Methanol** | **Water** |  |
| **HOMO energy (**$E_{HOMO}$**, eV)** | -5.388 | -5.433 | -5.440 | -5.466 |  |
| **LUMO energy (**$E_{LUMO}$**, eV)** | -1.343 | -1.397 | -1.394 | -1.400 |  |
| **Gap energy (**$\Delta E_{GAP},$**eV)** | 4.045 | 4.036 | 4.046 | 4.066 |  |
| **Ionization Energy (**$IE$**, eV)** | 5.388 | 5.433 | 5.440 | 5.466 |  |
| **Electronic Affinity (**$EA,$**eV)** | 1.343 | 1.397 | 1.394 | 1.400 |  |
| **Eletronegativity (**$\chi,$**eV)** | 3.366 | 3.415 | 3.417 | 3.433 |  |
| **Global Chemical Hardness (**$\eta,$**eV)** | 2.023 | 2.018 | 2.023 | 2.033 |  |
| **Global Chemical Softness (**$S,$ **eV^-1^)** | 0.494 | 0.496 | 0.494 | 0.492 |  |
| **Global Electrophilicity Index (**$\omega,$**eV)** | 2.800 | 2.890 | 2.886 | 2.899 |  |
| **Global Nucleophilicity Index (**$\epsilon,$**eV^-1^)** | 0.357 | 0.346 | 0.347 | 0.345 |  |
| **CCB3** | | | | | |
| **Descriptos** | **Chloroform** | **DMSO** | **Methanol** | **Water** |  |
| **HOMO energy** $E_{HOMO}$**(eV)** | -5.465 | -5.522 | -5.519 | -5.526 |  |
| **LUMO energy** $E_{LUMO}$**(eV)** | -1.230 | -1.288 | -1.284 | -1.291 |  |
| **Gap energy (**$\Delta E_{GAP},$**eV)** | 4.235 | 4.234 | 4.235 | 4.235 |  |
| **Ionization Energy (**$IE$**, eV)** | 5.465 | 5.522 | 5.519 | 5.526 |  |
| **Electronic Affinity (**$EA,$**eV)** | 1.230 | 1.288 | 1.284 | 1.291 |  |
| **Eletronegativity (**$\chi,$**eV)** | 3.348 | 3.405 | 3.402 | 3.409 |  |
| **Global Chemical Hardness (**$\eta,$**eV)** | 2.118 | 2.117 | 2.118 | 2.118 |  |
| **Global Chemical Softness (**$S,$ **eV^-1^)** | 0.472 | 0.472 | 0.472 | 0.472 |  |
| **Global Electrophilicity Index (**$\omega,$**eV)** | 2.646 | 2.738 | 2.732 | 2.743 |  |
| **Global Nucleophilicity Index (**$\epsilon,$**eV^-1^)** | 0.378 | 0.365 | 0.366 | 0.365 |  |
| **CCB4** | | | | | |
| **Descriptos** | **Chloroform** | **DMSO** | **Methanol** | **Water** |  |
| **HOMO energy** $E_{HOMO}$**(eV)** | -5.639 | -5.699 | -5.696 | -5.703 |  |
| **LUMO energy** $E_{LUMO}$**(eV)** | -1.385 | -1.448 | -1.444 | -1.452 |  |
| **Gap energy (**$\Delta E_{GAP},$**eV)** | 4.254 | 4.251 | 4.252 | 4.251 |  |
| **Ionization Energy (**$IE$**, eV)** | 5.639 | 5.699 | 5.696 | 5.703 |  |
| **Electronic Affinity (**$EA,$**eV)** | 1.385 | 1.448 | 1.444 | 1.452 |  |
| **Eletronegativity (**$\chi,$**eV)** | 3.512 | 3.574 | 3.570 | 3.578 |  |
| **Global Chemical Hardness (**$\eta,$**eV)** | 2.127 | 2.126 | 2.126 | 2.126 |  |
| **Global Chemical Softness (**$S,$ **eV^-1^)** | 0.470 | 0.470 | 0.470 | 0.470 |  |
| **Global Electrophilicity Index (**$\omega,$**eV)** | 2.899 | 3.004 | 2.997 | 3.011 |  |
| **Global Nucleophilicity Index (**$\epsilon,$**eV^-1^)** | 0.345 | 0.333 | 0.334 | 0.332 |  |

Supplementary Figure S1. Structures optimized in CHCl_3_, DMSO and H_2_O environments.


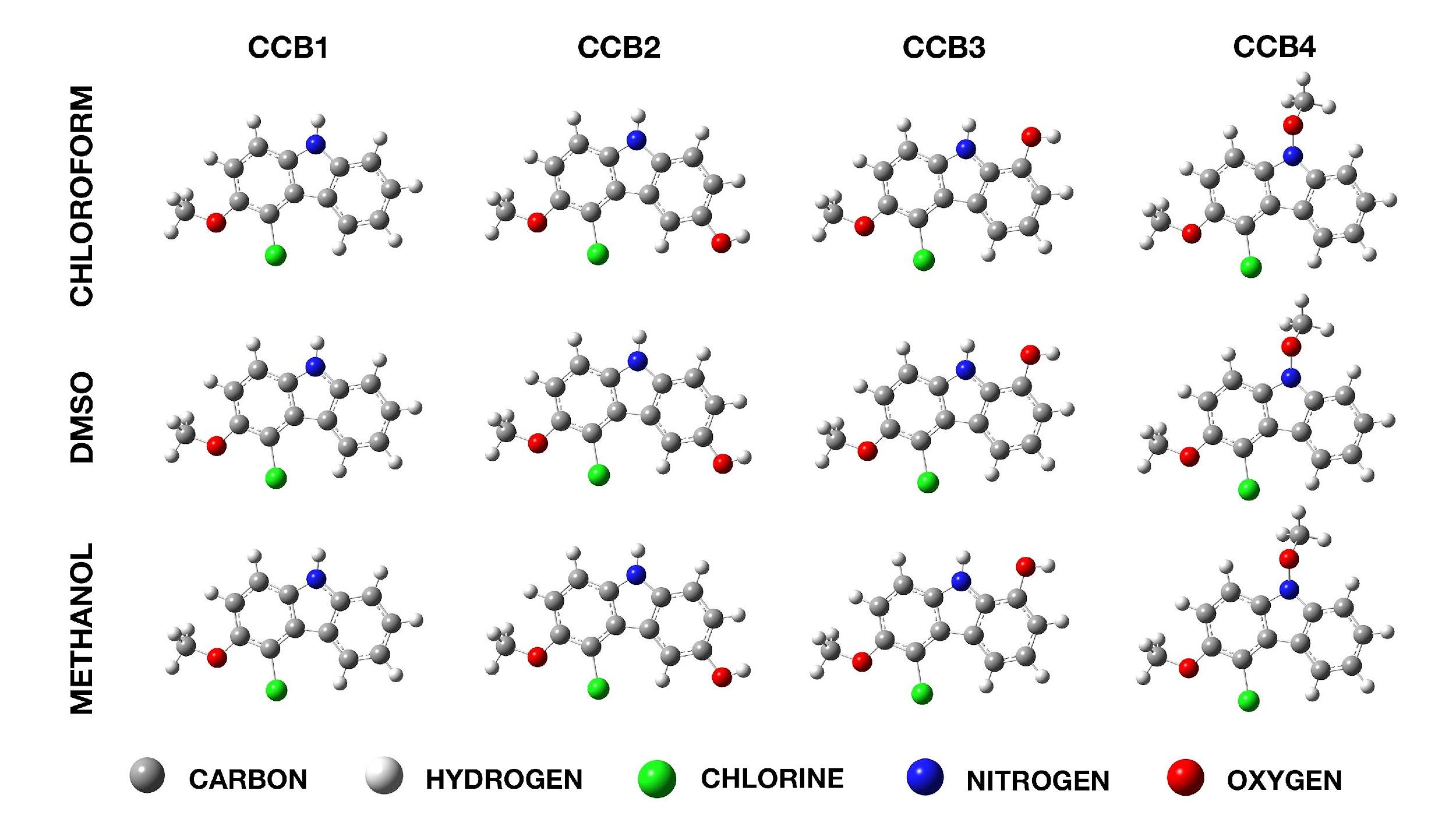
Table X1. Calculated chemical descriptors for CCB1–4 in chloroform, DMSO, methanol, and water using the B3LYP/6-311++G(d,p) method.

CCB1

Supplementary Figure S2. HOMO and LUMO molecular orbitals in the other solvents for CCB1.


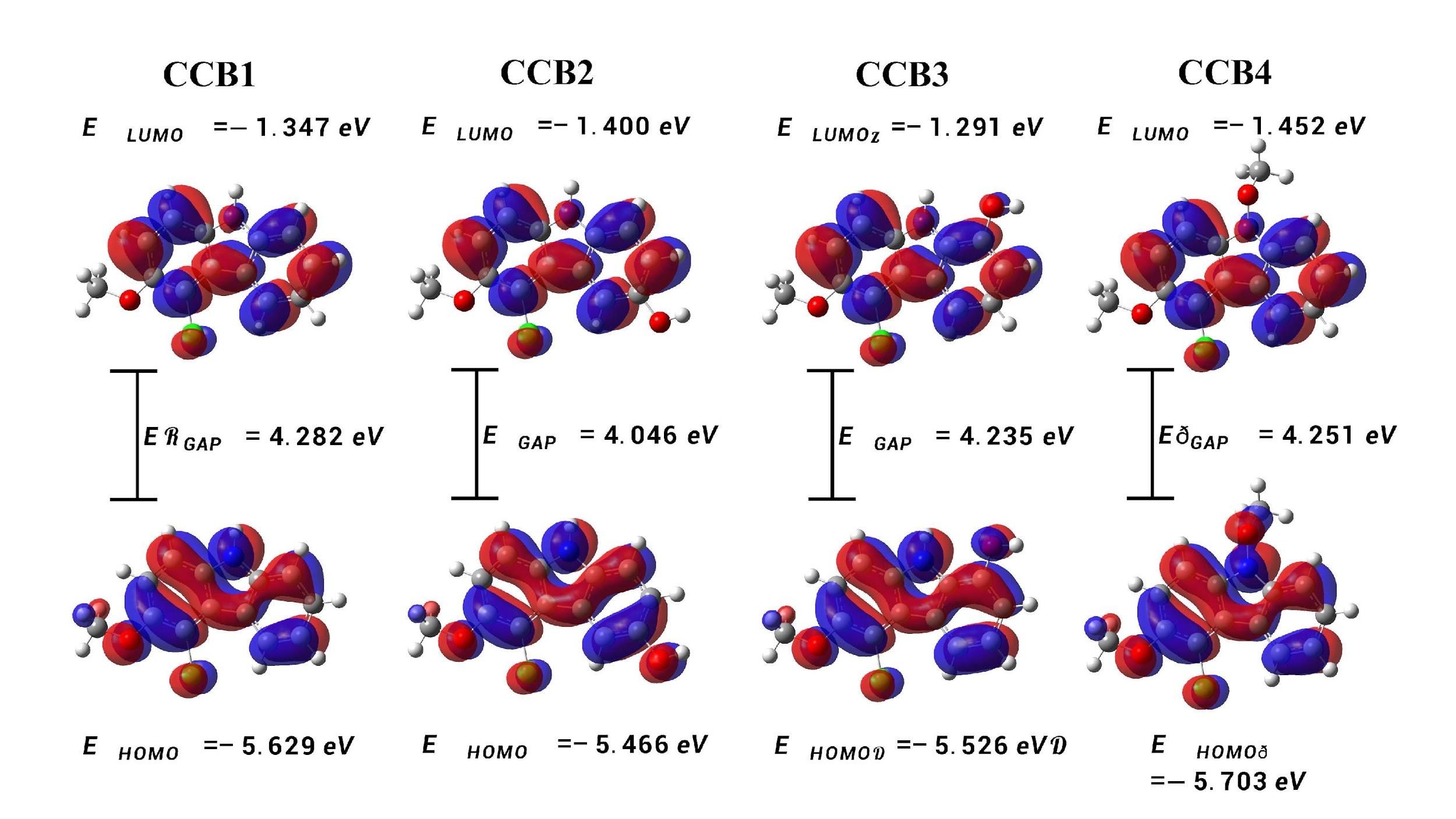


Supplementary Figure S3. HOMO and LUMO molecular orbitals in the other solvents for CCB2.


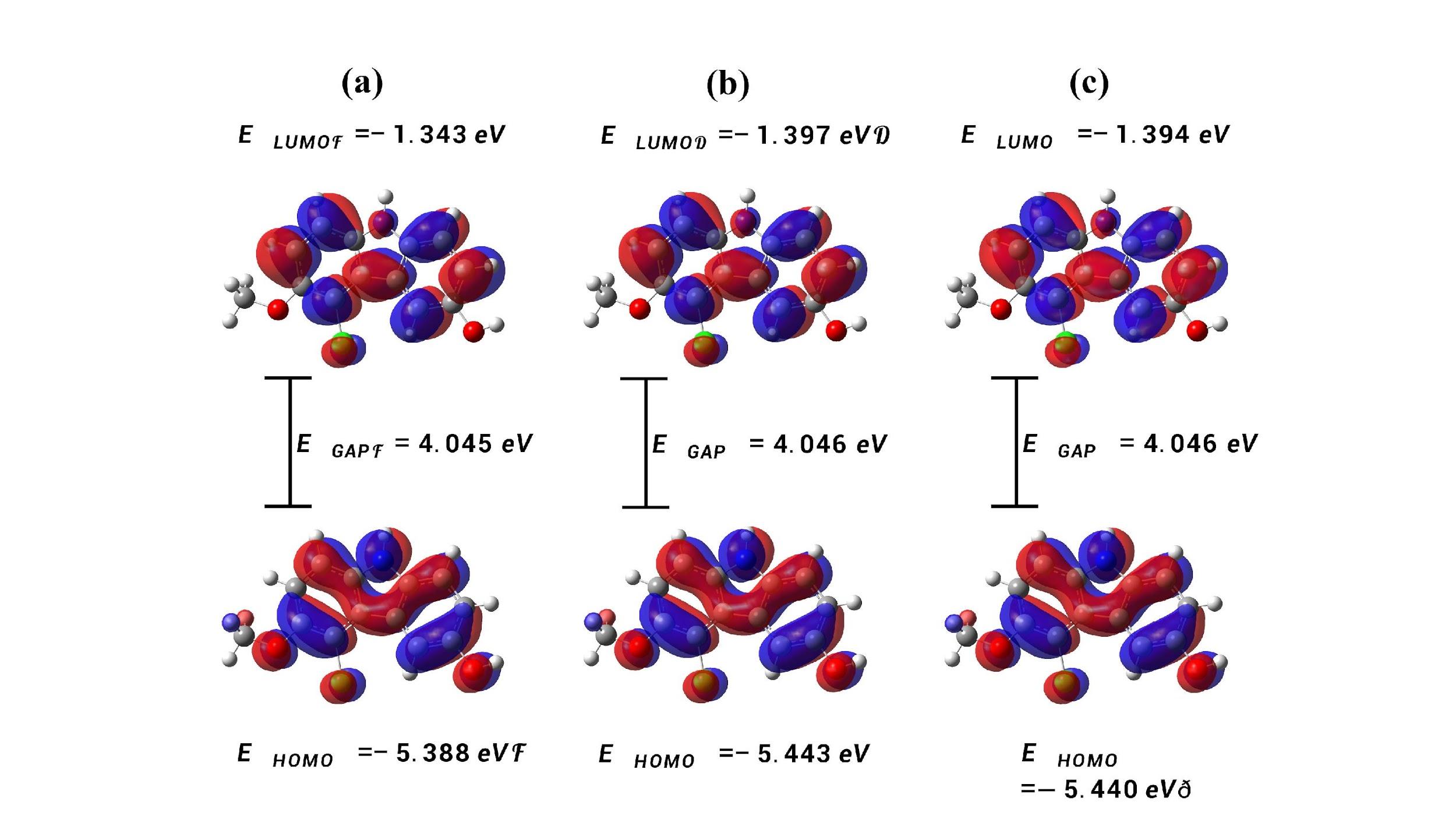


Supplementary Figure S4. HOMO and LUMO molecular orbitals in the other solvents for CCB3.


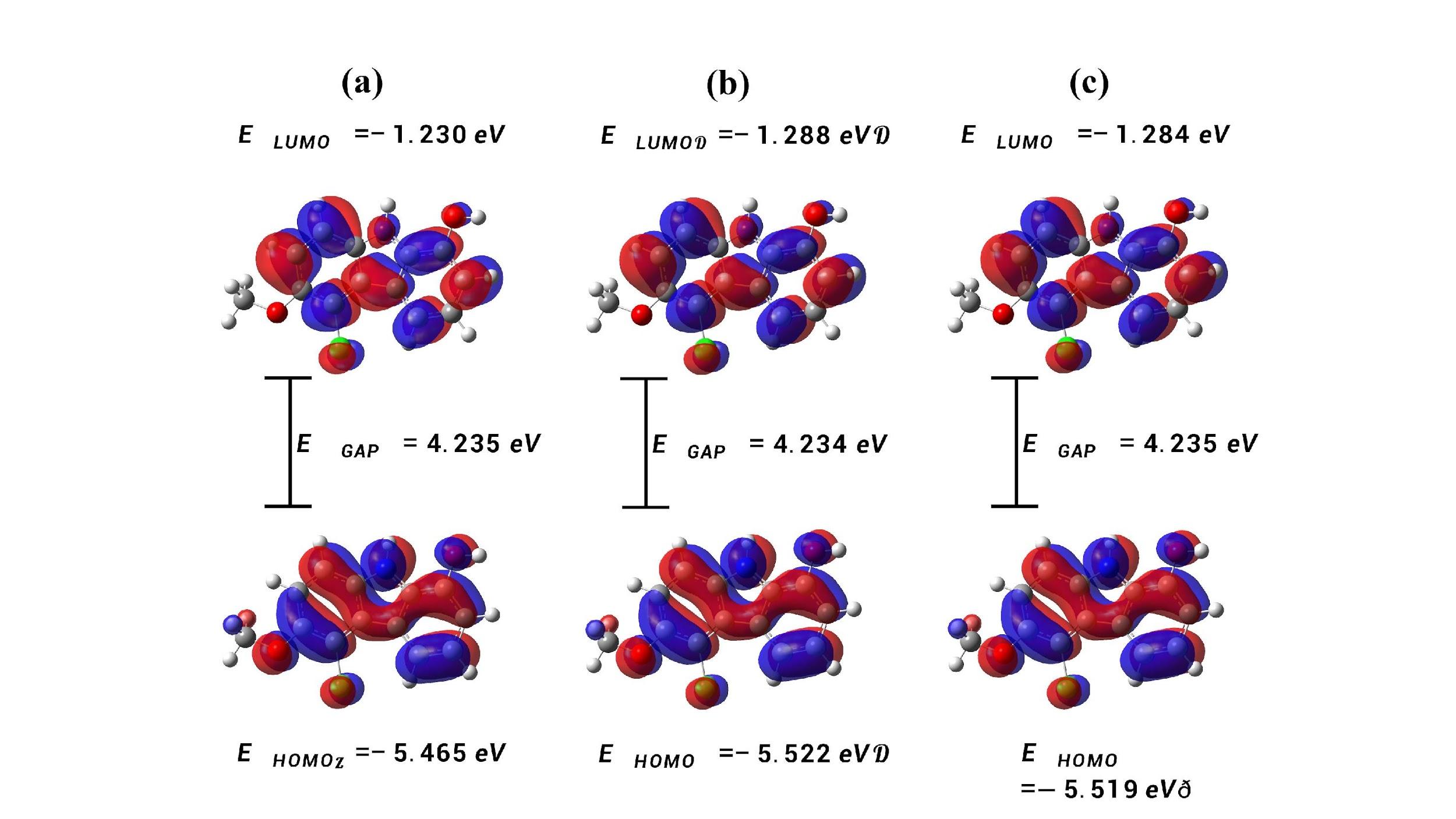


Supplementary Figure S5. HOMO and LUMO molecular orbitals in the other solvents for CCB4.


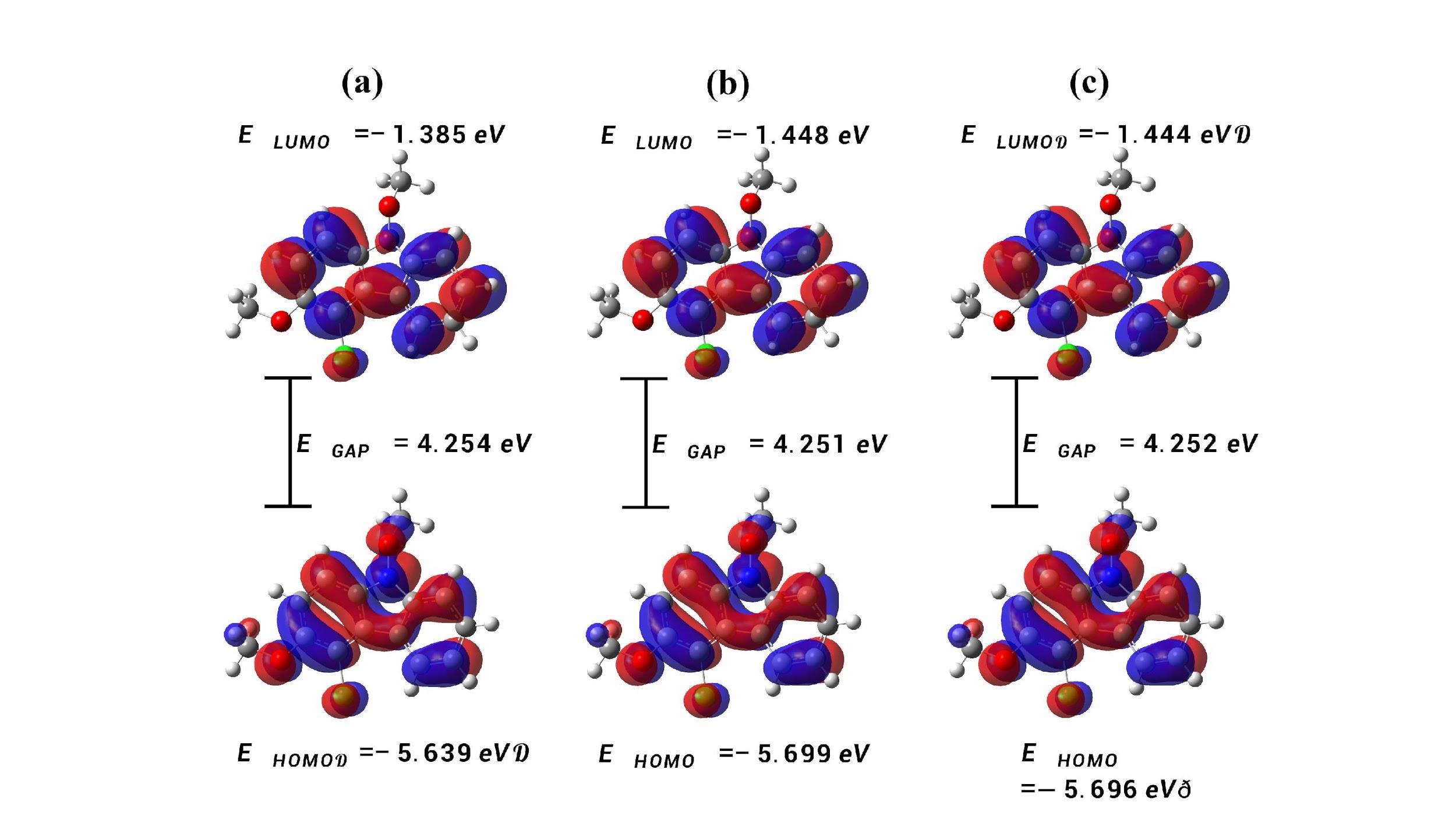


Supplementary Figure S6. Multiphilic descriptor values ​​for each atom of the CCB1 derivative.


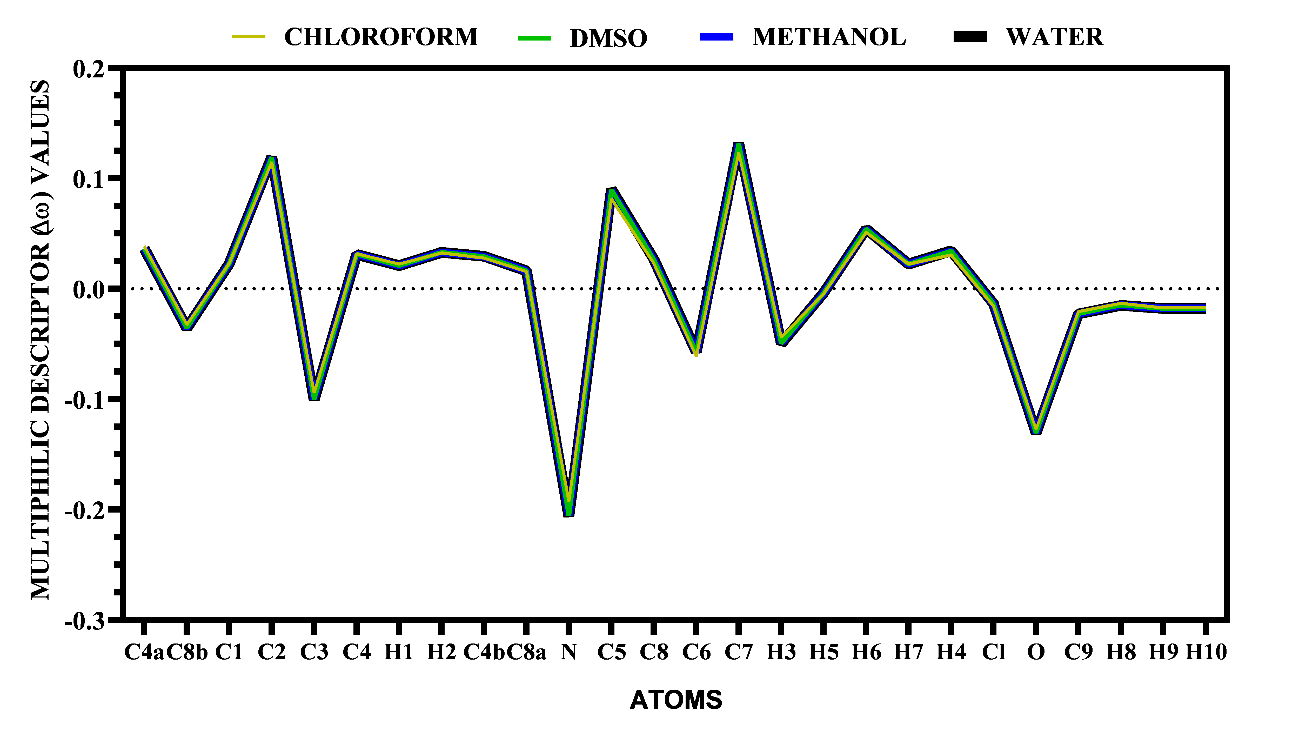


Supplementary Figure S7. Multiphilic descriptor values ​​for each atom of the CCB2 derivative.


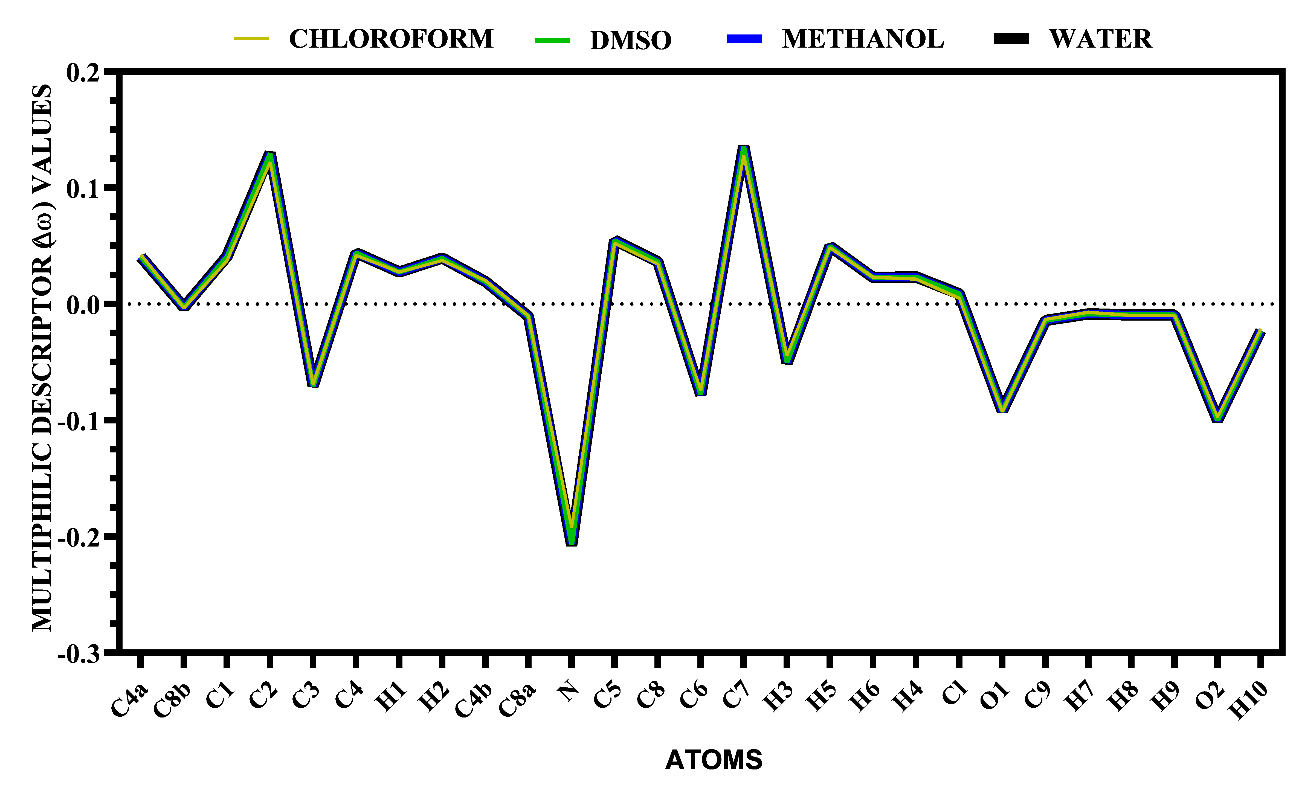


Supplementary Figure S8. Multiphilic descriptor values ​​for each atom of the CCB3 derivative.


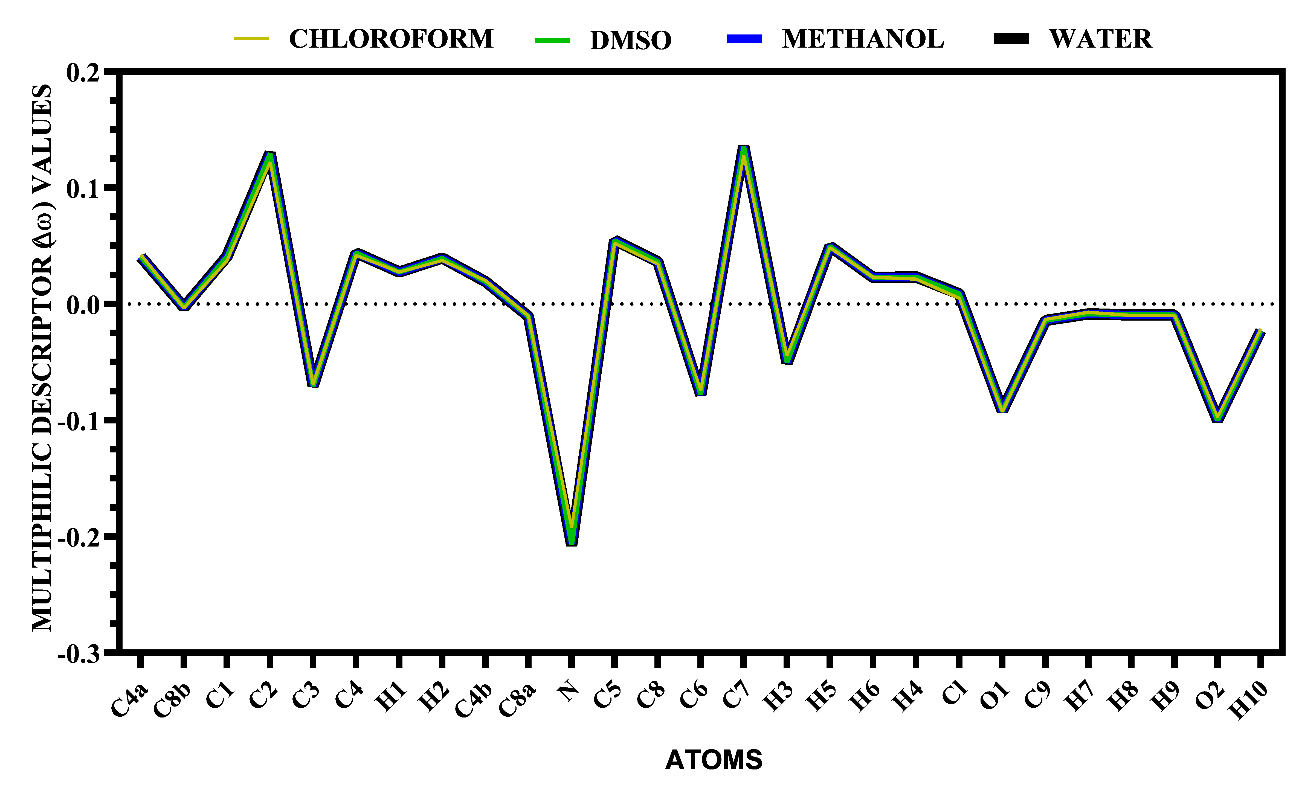


Supplementary Figure S9. Multiphilic descriptor values ​​for each atom of the CCB3 derivative.


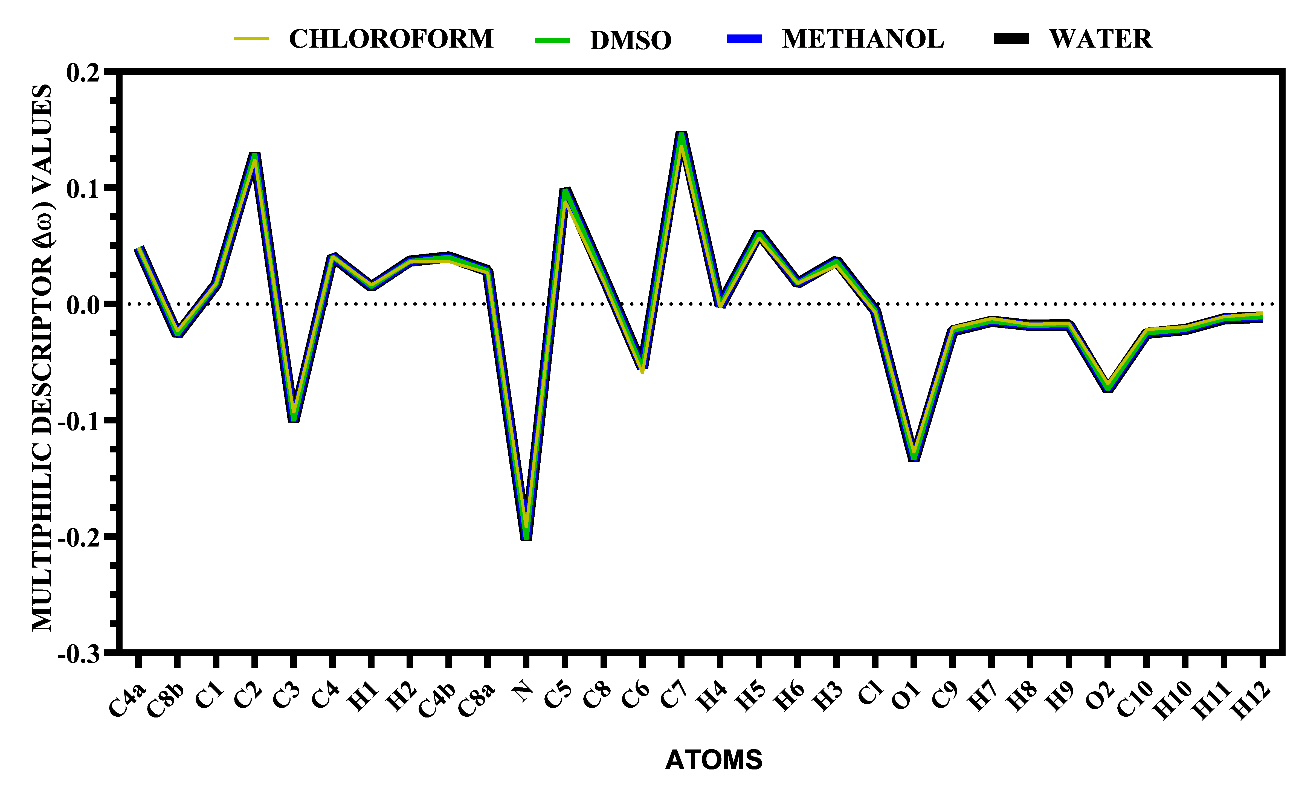


Supplementary Figure S10. MEP in the other solvents for the CCB1 and CCB2 derivatives.


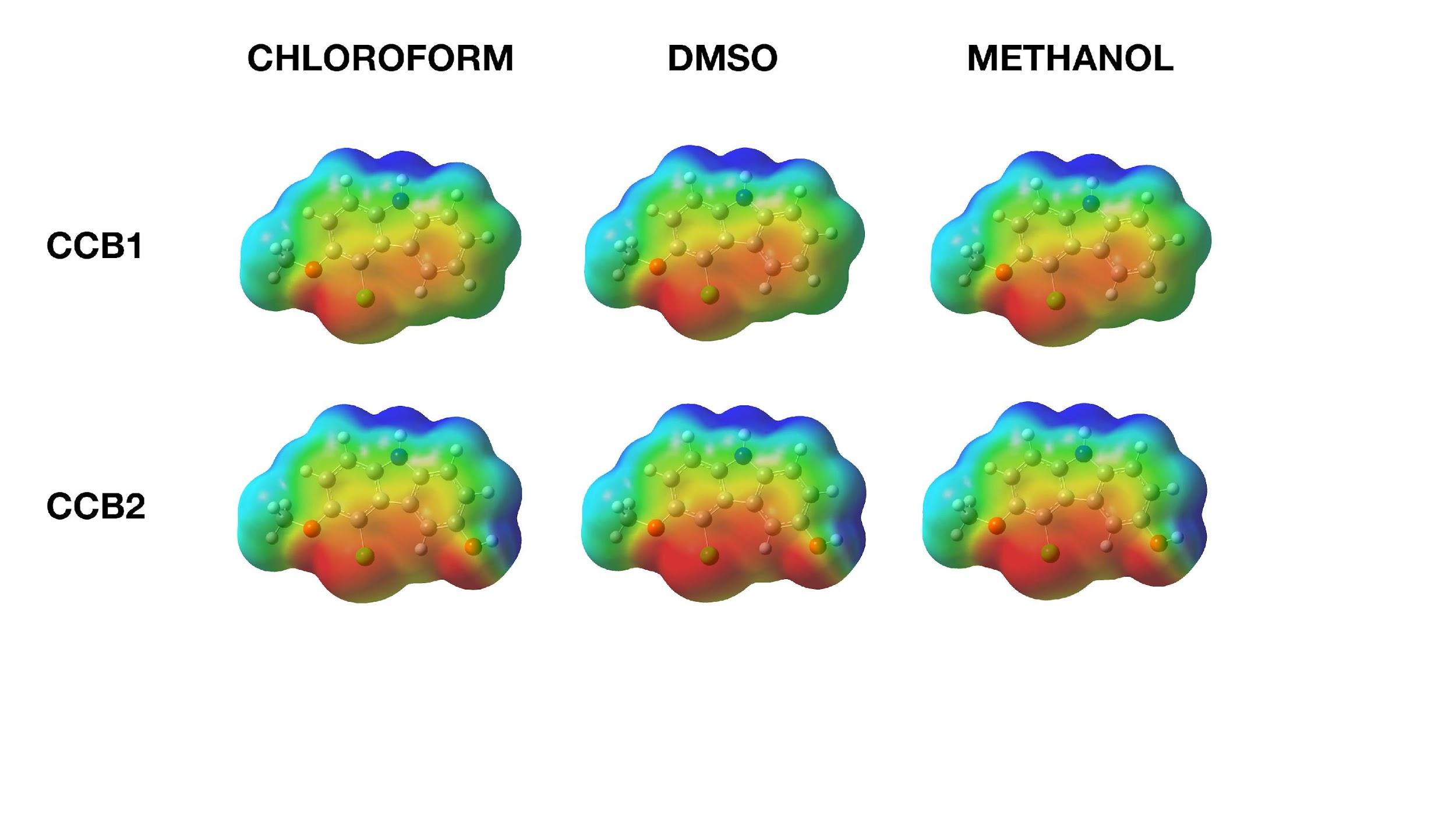


Supplementary Figure S11. MEP in the other solvents for the CCB3 and CCB4 derivatives.


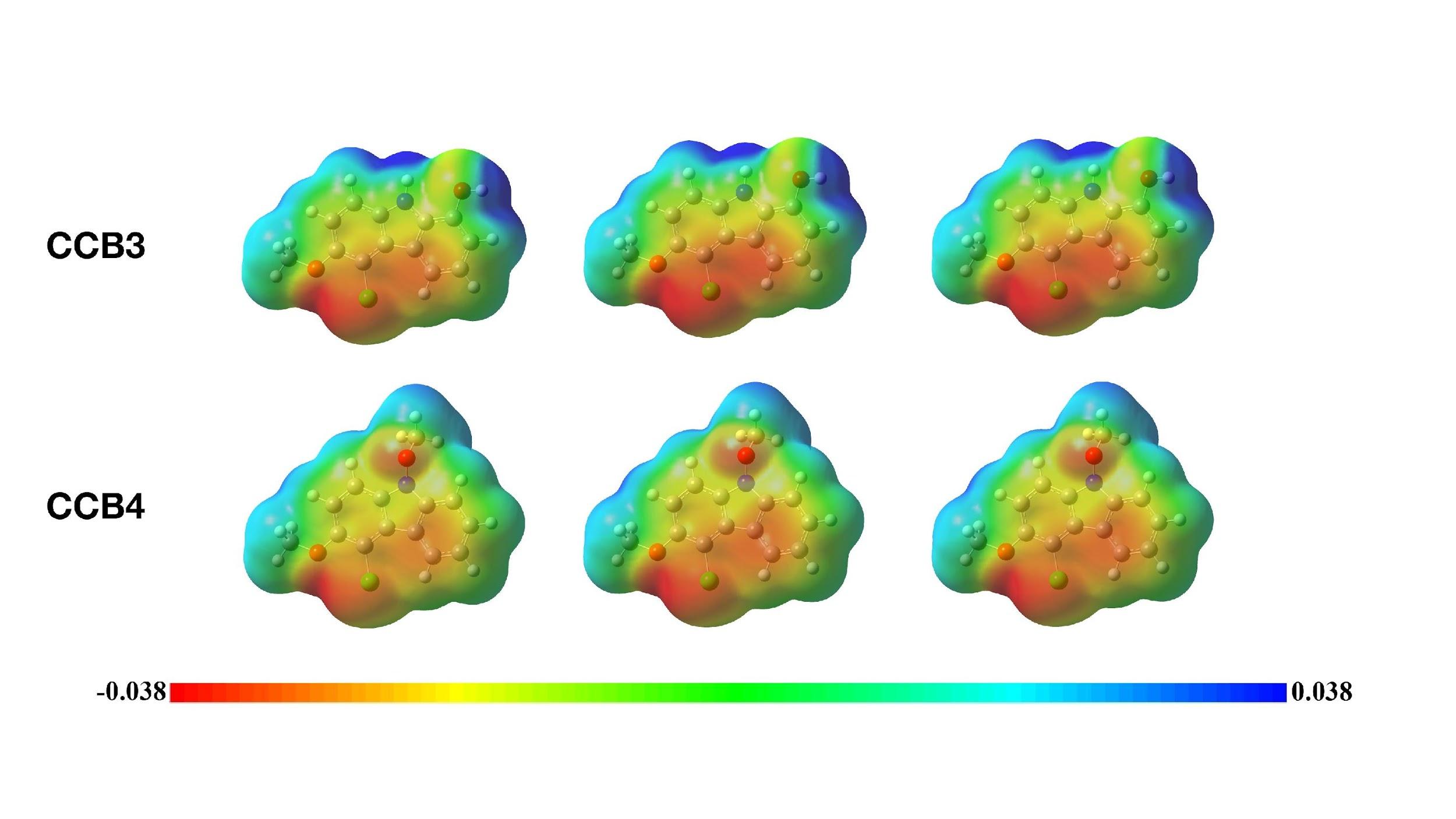

Supplement: Supplementary file 1 — Supplementary Material 1 [file 11030_2025_11446_MOESM1_ESM.docx]
